# New 1*E*,1′*E*-hydrazine-bis(phenoxy-1,2,3-triazol-acetamide) derivatives as potent inhibitors against acetylcholinesterase, butyrylcholinesterase, and α-glucosidase

**DOI:** 10.1039/d5ra03877d

**Published:** 2025-08-22

**Authors:** Shahab Kermaninia, Maryam Mohammadi-Khanaposhtani, Halil Şenol, Fatemeh Sadat Khajeh Mohammadilar, Navid Dastyafteh, Fatemeh Moradkhani, Saeedeh Saeedi, Bagher Larijani, Armin Dadgar, Aydın Aktaş, Nastaran Sadeghian, Parham Taslimi, Mohammad Mahdavi

**Affiliations:** a Endocrinology and Metabolism Research Center, Endocrinology and Metabolism Clinical Sciences Institute, Tehran University of Medical Sciences Tehran Iran momahdavi@tums.ac.ir; b Cellular and Molecular Biology Research Center, Health Research Institute, Babol University of Medical Sciences Babol Iran; c Department of Pharmaceutical Chemistry, Faculty of Pharmacy, Bezmialem Vakif University 34093 Fatih Istanbul Turkey; d Pharmaceutical and Heterocyclic Compounds Research Laboratory, Department of Chemistry, Iran University of Science and Technology Tehran 16846-13114 Iran; e Metabolic Disorders Research Center, Endocrinology and Metabolism Molecular-Cellular Sciences Institute, Tehran University of Medical Science Tehran Iran; f Drug Design and Development Research Center, The Institute of Pharmaceutical Sciences (TIPS), Tehran University of Medical Sciences Tehran Iran; g Vocational School of Health Service, Inonu University Malatya Turkey; h Department of Biotechnology, Faculty of Science, Bartin University Bartin Turkey parham_taslimi_un@yahoo.com

## Abstract

In this study, novel 1*E*,1′*E*-hydrazine-bis(phenoxy-1,2,3-triazol-acetamide) derivatives 10a–n were synthesized, and because of their structural features, they were evaluated against acetylcholinesterase (AChE), butyrylcholinesterase (BChE), and α-glucosidase. AChE and BChE are two important targets in the treatment of Alzheimer's disease (AD), and α-glucosidase is a carbohydrate-hydrolyzing enzyme with therapeutic importance in diabetes. Furthermore, cell studies were performed on the title compounds against SH-SY5Y neuroblastoma cells as a cancer cell line and HEK293 cells as a normal cell line. *In vitro* enzymatic evaluations demonstrated that these new compounds were active against the studied enzymes in comparison to standard inhibitors. In this regard, all the synthesized compounds were more potent than the standard inhibitors tacrine and donepezil against BChE, and most of these compounds were more potent than tacrine against AChE. Moreover, most of the target synthesized compounds were more potent than the standard inhibitor acarbose against α-glucosidase. The most potent compound against AChE and BChE was the 2,4-dichloro derivative 10k, and the most potent compound against α-glucosidase was the 2-chloro derivative 10h. Moreover, *in vitro* cell studies demonstrated that compounds 10k and 10h with a selectivity index of >10 demonstrated more cytotoxic effects on the cancer cell line SH-SY5Y than on the normal cell line HEK293. A docking study showed that the latter compounds attached to the active sites of the target enzymes with binding energies more favorable than those of the selected standard inhibitors. Furthermore, docking studies demonstrated that compound 10k interacted with both the catalytic and peripheral anionic sites of AChE and BChE. This property led to the better efficacy of the compound in the treatment of AD.

## Abstract 2

In this study, novel 1*E*,1′*E*-hydrazine-bis(phenoxy-1,2,3-triazol-acetamide) derivatives 10a–n were synthesized, and because of their structural features, they were evaluated against acetylcholinesterase, butyrylcholinesterase, and α-glucosidase.

## Graphical abstract

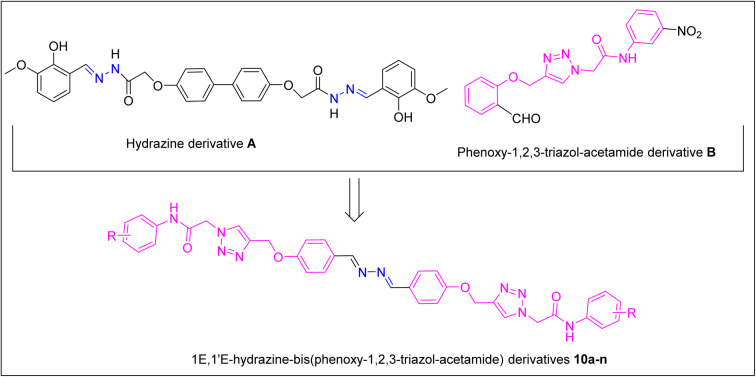

## Introduction

Alzheimer's disease (AD), as the most common cause of death in elderly populations, is the most common type of neurodegenerative disorder.^1^ As this disease progresses, brain neurons are destroyed; therefore, control of this disease has a great importance. During the pathological progression of AD in the brain, cholinergic terminals deteriorate, the aggregation of β-amyloid (Aβ) plaques occurs, oxidative stress increases, and neurofibrillary tangles form.^2–4^ As cholinergic terminals in the brain deteriorate, production of acetylcholine (ACh) decreases and cognitive impairment increases. ACh is an important neurotransmitter in the memory-related processes of the brain.^5^ The most important enzyme that degrades ACh and terminates its action in cholinergic terminals is acetylcholinesterase (AChE).^6^ Inhibition of AChE leads to an increase in the ACh level, and this mechanism is a palliative treatment for AD.^7^ Therefore, for many years, the most widely used drugs to control AD have been AChE inhibitors.^8^ Another ACh-degrading enzyme is butyrylcholinesterase (BChE), which is found in plasma.^9^ Recently, several credible reports have shown that in AD, AChE activity reduces in specific regions of the brain while BChE activity increases in these regions.^10^ According to the mentioned points, inhibition of AChE and BChE improve the symptoms of AD.^11^

X-ray crystallographic structures of ChEs showed that these hydrolyzing enzymes have four components in their binding sites with known inhibitors: (1) a catalytic anionic site (CAS) with a catalytic triad (CT) and anionic site (AS) as sub-components, (2) a peripheral anionic site (PAS), (3) an acyl pocket (AP), and (4) an oxyanion hole (OAH).^12^ Interaction of a compound with the CAS component is necessary for the inhibitory effect against ChEs. On the other hand, several studies showed that the PAS area of AChE could accelerate the polymerization of Aβ peptides, and its inhibition disrupted polymerization of these peptides.^13^ Therefore, interaction of the ChE inhibitors with the PAS component is also valuable for the management of AD.^14^ According to the presented points, the design of new ChE inhibitors with inhibitory activities against both AChE and BChE that interact with the PAS component of the binding sites of these enzymes has become particularly important for medicinal chemists.^15^

Molecular hybridization theory (MHT), which is based on the combination of bioactive pharmacophores of potent bioactive molecules, has recently gained particular importance in the design of lead drug compounds. Using MHT, recently our research group introduced a very new series of derivatives with anti-AD, anti-diabetic, and anti-cancer activities.^16–18^

**Fig. 1:**
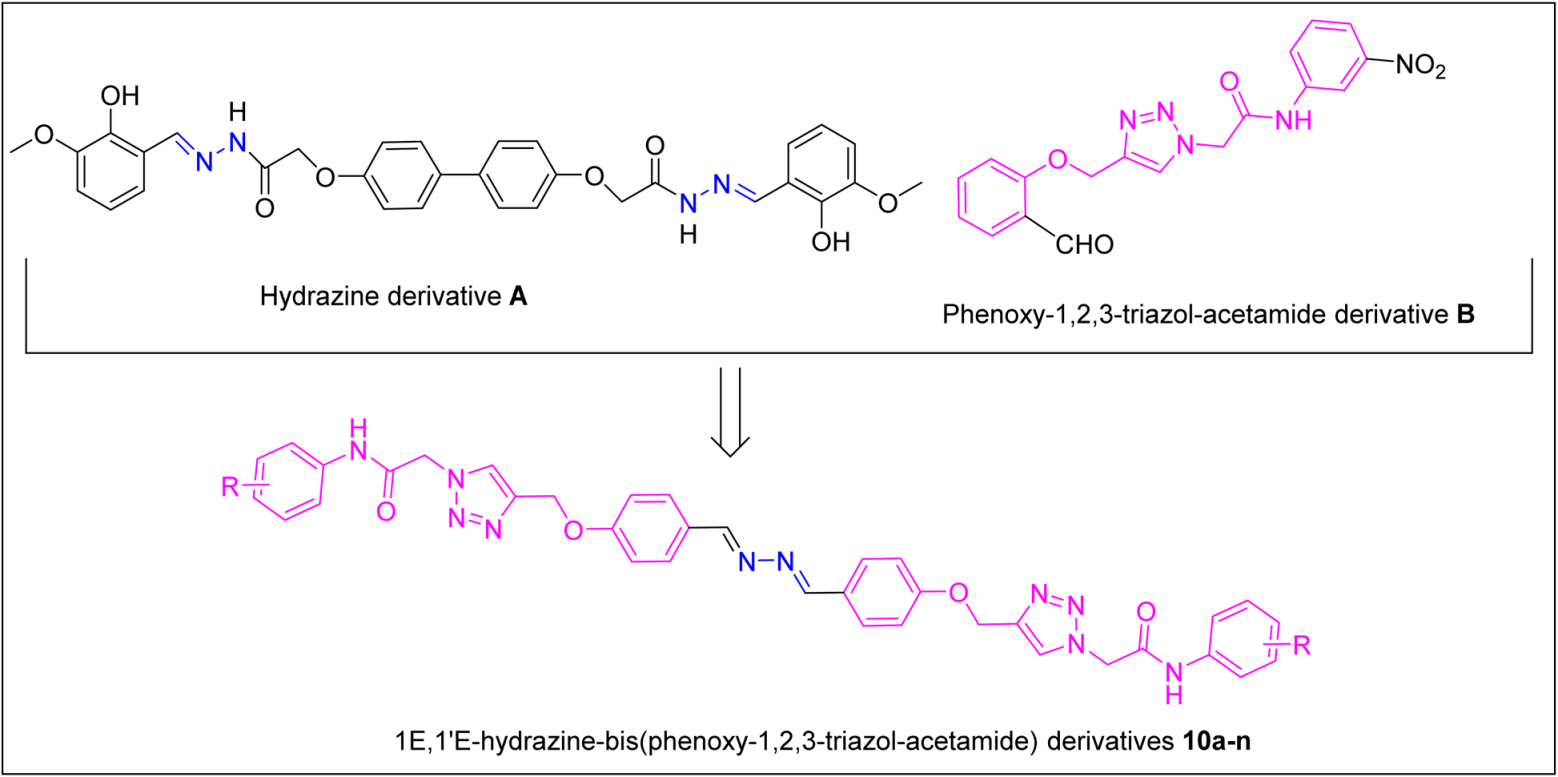
Design strategy for derivatives 10a–n as new ChE inhibitors.

Schiff basses are popular linkers for binding bioactive pharmacophores because they have various biological effects.^19^ In this regard, Ibrahim *et al.* used Schiff bases for the synthesis of novel bis-acylhydrazones of 2,2′-(1,1′-biphenyl)-4,4′-diylbis(oxy)di(acetohydrazide) derivatives as dual inhibitors against AChE and BChE. Among these compounds, derivative A was the most potent compound (Fig. 1).^20^ We and other research groups have also reported on a series of hybrid molecules containing a 1,2,3-triazole ring with high inhibitory activities against ChEs.^16^ For instance, the phenoxy-1,2,3-triazol-acetamide derivative B was a potent inhibitor against AChE (Fig. 1).^21^

Herein, we used the (1*E*,1′*E*)-hydrazine linker as a Schiff base and attached two phenoxy-1,2,3-triazole-*N*-phenylacetamide moieties for the design of 1*E*,1′*E*-hydrazine-bis(phenoxy-1,2,3-triazol-acetamide) derivatives 10a–n as new ChE inhibitors (Fig. 1). After synthesis, these compounds were evaluated against AChE and BChE *in vitro* and *in silico*.

**Fig. 2:**
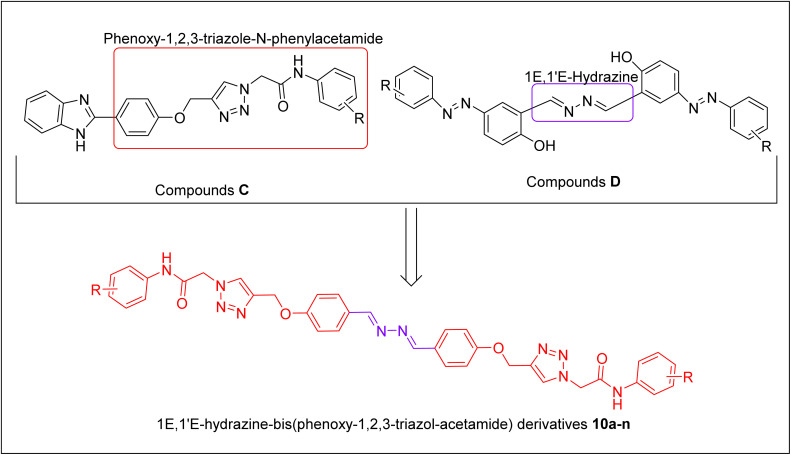
Design strategy for derivatives 10a–n as new α-glucosidase inhibitors.

α-Glucosidase is an important digestive enzyme that is located in the small intestine, and is responsible for the breakdown of disaccharides and oligosaccharide to glucose. Glucose is easily absorbed from the intestine and enters the bloodstream.^22^ Therefore, α-glucosidase inhibition is an important therapeutic target for carbohydrate-related diseases such as type 2 diabetes, cancer, and viral infections.^23^ Recently, our research team introduced several series of phenoxy-1,2,3-triazole-*N*-phenylacetamide derivatives as α-glucosidase inhibitors by MHT.^24^ For example, benzimidazole-phenoxy-1,2,3-triazole-*N*-phenylacetamide derivatives C showed high inhibitory activity against α-glucosidase.^25^ On the other hand, (1*E*,1′*E*)-hydrazine as a linker was used in the design of α-glucosidase inhibitors D.^26^ Therefore, we also evaluated 1*E*,1′*E*-hydrazine-bis(phenoxy-1,2,3-triazol-acetamide) derivatives 10a–n against α-glucosidase (Fig. 2).

## Materials and methods

### Synthesis of 4,4′-((1*E*,1′*E*)-hydrazine-1,2-diylidenebis(methaneylylidene))diphenol (3)

A solution of 4-hydroxybenzaldehyde (1, 20 mmol) and hydrazine (2, 10 mmol) in ethanol was stirred at 50 °C. After 24 h, water was added to the reaction mixture, and the observed participate was separated by filtration to give pure 4,4′-((1*E*,1′*E*)-hydrazine-1,2-diylidenebis(methaneylylidene))diphenol (3).

### Synthesis of 1,2-bis((*E*)-4-(prop-2-yn-1-yloxy)benzylidene)hydrazine (5)

A mixture of compound 3 (10 mmol), propargyl bromide (4) (20 mmol), and K_2_CO_3_ (30 mmol) in DMF was stirred at 60 °C for 18 h. After that, water was added to the reaction mixture and the observed participate was separated by filtration. The residue was dried in 60 °C to give pure 1,2-bis((*E*)-4-(prop-2-yn-1-yloxy)benzylidene)hydrazine (5).

### General synthesis of 1,2-bis((*E*)-4-(prop-2-yn-1-yloxy)benzylidene)hydrazine derivatives 10a–n

Compounds 9a–n were prepared *in situ* according to our previous works.^25^ To a stirred reaction mixture containing the prepared compounds 9a–n (2 mmol), compound 5 (1 mmol), sodium ascorbate (10 mol%), and CuSO_4_·5H_2_O (5 mol%) were added. The obtained mixture was then stirred at room temperature for 48 h. After that, the latter reaction mixture was poured into crushed ice. The precipitated products were then filtered off, washed with cold water, and purified by chromatography on silica gel using petroleum ether/ethyl acetate (4 : 1) to give the target compounds 10a–n.

#### 2,2′-((((((1*E*,1′*E*)-Hydrazine-1,2-diylidenebis(methaneylylidene))bis(4,1-phenylene))bis(oxy))bis(methylene))bis(1*H*-1,2,3-triazole-4,1-diyl))bis(*N*-(*o*-tolyl)acetamide) (10a)

Cream solid; yield: 85%; MP = 195–197 °C; ^1^H NMR (400 MHz, DMSO-*d*_6_) *δ* 9.83 (s, 2H), 8.67 (s, 2H), 8.31 (s, 2H), 7.84 (d, *J* = 8.6 Hz, 4H), 7.45 (d, *J* = 8.6 Hz, 2H), 7.25 (d, *J* = 8.2 Hz, 2H), 7.22–7.17 (m, 6H), 7.13 (d, *J* = 7.2 Hz, 2H), 5.43 (s, 4H), 5.28 (s, 4H), 2.25 (s, 6H); ^13^C NMR (101 MHz, DMSO-*d*_6_) *δ* 164.85, 161.01, 160.95, 142.59, 135.98, 132.06, 130.93, 130.47, 127.32, 126.95, 126.56, 126.05, 125.21, 115.60, 61.65, 52.42, 18.29; anal. calcd: C_38_H_36_N_10_O_4_; C, 65.50; H, 5.21; N, 20.10; found C, 65.68; H, 5.40; N, 20.29.

#### 2,2′-((((((1*E*,1′*E*)-Hydrazine-1,2-diylidenebis(methaneylylidene))bis(4,1-phenylene))bis(oxy))bis(methylene))bis(1*H*-1,2,3-triazole-4,1-diyl))bis(*N*-(*m*-tolyl)acetamide) (10b)

Cream solid; yield: 84%; MP = 205–207 °C; ^1^H NMR (400 MHz, DMSO-*d*_6_) *δ* 10.43 (s, 2H), 8.67 (s, 2H), 8.31 (s, 2H), 7.85 (d, *J* = 8.7 Hz, 4H), 7.44 (s, 2H), 7.38 (d, *J* = 8.2 Hz, 2H), 7.26–7.18 (m, 6H), 6.92 (d, *J* = 7.5 Hz, 2H), 5.37 (s, 4H), 5.28 (s, 4H), 2.29 (s, 6H); ^13^C NMR (101 MHz, DMSO-*d*_6_) *δ* 164.58, 161.02, 160.96, 142.59, 138.81, 138.62, 130.48, 129.24, 127.33, 126.96, 124.97, 120.23, 116.88, 115.60, 61.65, 52.72, 21.65; anal. calcd: C_38_H_36_N_10_O_4_; C, 65.50; H, 5.21; N, 20.10; found C, 65.66; H, 5.42; N, 20.31.

#### 2,2′-((((((1*E*,1′*E*)-Hydrazine-1,2-diylidenebis(methaneylylidene))bis(4,1-phenylene))bis(oxy))bis(methylene))bis(1*H*-1,2,3-triazole-4,1-diyl))bis(*N*-(*p*-tolyl)acetamide) (10c)

Cream solid; yield: 86%; MP = 222–224 °C; ^1^H NMR (400 MHz, DMSO-*d*_6_) *δ* 10.42 (s, 2H), 8.67 (s, 2H), 8.31 (s, 2H), 7.85 (d, *J* = 8.7 Hz, 4H), 7.48 (d, *J* = 8.3 Hz, 4H), 7.20 (d, *J* = 8.6 Hz, 4H), 7.15 (d, *J* = 8.3 Hz, 4H), 5.35 (s, 4H), 5.28 (s, 4H), 2.27 (s, 6H); ^13^C NMR (101 MHz, DMSO-*d*_6_) *δ* 164.38, 161.01, 160.95, 142.58, 136.37, 133.24, 130.47, 129.78, 127.32, 126.95, 119.69, 115.60, 61.65, 52.68, 20.93; anal. calcd: C_38_H_36_N_10_O_4_; C, 65.50; H, 5.21; N, 20.10; found C, 65.69; H, 5.40; N, 20.27.

#### 2,2′-((((((1*E*,1′*E*)-Hydrazine-1,2-diylidenebis(methaneylylidene))bis(4,1-phenylene))bis(oxy))bis(methylene))bis(1*H*-1,2,3-triazole-4,1-diyl))bis(*N*-(2,3-dimethylphenyl)acetamide) (10d)

Cream solid; yield: 72%; MP = 227–229 °C; ^1^H NMR (400 MHz, DMSO-*d*_6_) *δ* 9.90 (s, 2H), 8.66 (s, 2H), 8.31 (s, 2H), 7.84 (d, *J* = 8.6 Hz, 4H), 7.26–7.12 (m, 6H), 7.07 (d, *J* = 8.3 Hz, 4H), 5.41 (s, 4H), 5.28 (s, 4H), 2.26 (s, 6H), 2.12 (s, 6H); ^13^C NMR (101 MHz, DMSO-*d*_6_) *δ* 164.88, 161.02, 160.95, 142.56, 137.64, 135.72, 131.53, 130.47, 127.77, 127.32, 126.94, 125.79, 123.74, 115.60, 61.65, 52.37, 20.62, 14.49; anal. calcd: C_40_H_40_N_10_O_4_; C, 66.28; H, 5.56; N, 19.32; found C, 66.45; H, 5.77; N, 19.50.

#### 2,2′-((((((1*E*,1′*E*)-Hydrazine-1,2-diylidenebis(methaneylylidene))bis(4,1-phenylene))bis(oxy))bis(methylene))bis(1*H*-1,2,3-triazole-4,1-diyl))bis(*N*-(2,4-dimethoxyphenyl)acetamide) (10e)

Brown solid; yield: 88%; MP = 241–243 °C; ^1^H NMR (400 MHz, DMSO-*d*_6_) *δ* 9.65 (s, 2H), 8.67 (s, 2H), 8.29 (s, 2H), 7.84 (d, *J* = 8.6 Hz, 4H), 7.73 (d, *J* = 8.8 Hz, 2H), 7.20 (d, *J* = 8.6 Hz, 4H), 6.66 (d, *J* = 2.4 Hz, 2H), 6.50 (dd, *J* = 8.8, 2.4 Hz, 2H), 5.42 (s, 4H), 5.27 (s, 4H), 3.86 (s, 6H), 3.76 (s, 6H); ^13^C NMR (101 MHz, DMSO-*d*_6_) *δ* 164.56, 161.02, 160.95, 157.51, 151.81, 142.56, 130.47, 127.32, 126.96, 123.84, 120.02, 115.60, 104.56, 99.36, 61.65, 56.23, 55.77, 52.55; anal. calcd: C_40_H_40_N_10_O_8_; C, 60.91; H, 5.11; N, 17.76; found C, 70.09; H, 5.30; N, 17.93.

#### 2,2′-((((((1*E*,1′*E*)-Hydrazine-1,2-diylidenebis(methaneylylidene))bis(4,1-phenylene))bis(oxy))bis(methylene))bis(1*H*-1,2,3-triazole-4,1-diyl))bis(*N*-(4-ethylphenyl)acetamide) (10f)

Cream solid; yield: 90%; MP = 201–203 °C; ^1^H NMR (400 MHz, DMSO-*d*_6_) *δ* 10.43 (s, 2H), 8.67 (s, 2H), 8.31 (s, 2H), 7.85 (d, *J* = 8.7 Hz, 4H), 7.50 (d, *J* = 8.3 Hz, 4H), 7.22–7.16 (m, 8H), 5.36 (s, 4H), 5.28 (s, 4H), 2.57 (q, *J* = 7.6 Hz, 4H), 1.17 (t, *J* = 7.5 Hz, 6H); ^13^C NMR (101 MHz, DMSO-*d*_6_) *δ* 164.39, 161.02, 160.95, 142.58, 139.68, 136.56, 130.47, 128.59, 127.33, 126.96, 119.77, 115.61, 61.65, 52.67, 28.06, 16.12; anal. calcd: C_40_H_40_N_10_O_4_; C, 66.28; H, 5.56; N, 19.32; found C, 66.49; H, 5.71; N, 19.50.

#### 2,2′-((((((1*E*,1′*E*)-Hydrazine-1,2-diylidenebis(methaneylylidene))bis(4,1-phenylene))bis(oxy))bis(methylene))bis(1*H*-1,2,3-triazole-4,1-diyl))bis(*N*-(2-fluorophenyl)acetamide) (10g)

Brown solid; yield: 83%; MP = 219–221 °C; ^1^H NMR (400 MHz, DMSO-*d*_6_) *δ* 10.36 (s, 2H), 8.67 (s, 2H), 8.31 (s, 2H), 7.97–7.89 (m, 2H), 7.85 (d, *J* = 8.6 Hz, 4H), 7.35–7.27 (m, 2H), 7.23–7.12 (m, 8H), 5.48 (s, 4H), 5.28 (s, 4H); ^13^C NMR (101 MHz, DMSO-*d*_6_) *δ* 165.30, 161.02, 160.95, 153.93 (d, *J* = 245.2 Hz), 142.62, 130.47, 127.33, 127.00, 126.18 (d, *J* = 7.5 Hz), 125.93 (d, *J* = 11.3 Hz), 125.01 (d, *J* = 3.5 Hz), 124.24, 116.11 (d, *J* = 19.3 Hz), 115.60, 61.65, 52.47; anal. calcd: C_36_H_30_F_2_N_10_O_4_; C, 61.36; H, 4.29; N, 19.88; found C, 61.52; H, 4.43; F, 5.39; N, 20.05.

#### 2,2′-((((((1*E*,1′*E*)-Hydrazine-1,2-diylidenebis(methaneylylidene))bis(4,1-phenylene))bis(oxy))bis(methylene))bis(1*H*-1,2,3-triazole-4,1-diyl))bis(*N*-(2-chlorophenyl)acetamide) (10h)

Yellow solid; yield: 76%; MP = 209–211 °C; ^1^H NMR (400 MHz, DMSO-*d*_6_) *δ* 10.80 (s, 2H), 8.66 (s, 2H), 8.29 (s, 2H), 8.00 (d, *J* = 8.0 Hz, 2H), 7.84 (d, *J* = 8.6 Hz, 4H), 7.79–7.69 (m, 4H), 7.44 (t, *J* = 7.1 Hz, 2H), 7.20 (d, *J* = 8.7 Hz, 4H), 5.47 (s, 4H), 5.28 (s, 4H); ^13^C NMR (101 MHz, DMSO-*d*_6_) *δ* 165.43, 161.01, 160.94, 142.95, 142.70, 134.66, 130.77, 130.47, 127.33, 126.96, 126.42, 126.04, 125.57, 115.60, 61.62, 52.42; anal. calcd: C_36_H_30_Cl_2_N_10_O_4_; C, 58.62; H, 4.10; N, 18.99; found C, 58.80; H, 4.31; N, 19.17.

#### 2,2′-((((((1*E*,1′*E*)-Hydrazine-1,2-diylidenebis(methaneylylidene))bis(4,1-phenylene))bis(oxy))bis(methylene))bis(1*H*-1,2,3-triazole-4,1-diyl))bis(*N*-(3-chlorophenyl)acetamide) (10i)

Yellow solid; yield: 83%; MP = 235–237 °C; ^1^H NMR (400 MHz, DMSO-*d*_6_) *δ* 10.12 (s, 2H), 8.67 (s, 2H), 8.32 (s, 2H), 7.84 (d, *J* = 8.7 Hz, 4H), 7.76 (d, *J* = 8.0 Hz, 2H), 7.54 (d, *J* = 7.9 Hz, 2H), 7.36 (t, *J* = 7.4 Hz, 2H), 7.25 (d, *J* = 7.5 Hz, 2H), 7.20 (d, *J* = 8.7 Hz, 4H), 5.49 (s, 4H), 5.28 (s, 4H); ^13^C NMR (101 MHz, DMSO-*d*_6_) *δ* 165.36, 161.02, 160.94, 142.63, 134.62, 130.47, 130.12, 128.06, 127.33, 127.23, 127.01, 126.77, 126.39, 115.60, 61.63, 52.42; anal. calcd: C_36_H_30_Cl_2_N_10_O_4_; C, 58.62; H, 4.10; N, 18.99; found C, 58.80; H, 4.31; N, 19.12.

#### 2,2′-((((((1*E*,1′*E*)-Hydrazine-1,2-diylidenebis(methaneylylidene))bis(4,1-phenylene))bis(oxy))bis(methylene))bis(1*H*-1,2,3-triazole-4,1-diyl))bis(*N*-(4-chlorophenyl)acetamide) (10j)

Yellow solid; yield: 90%; MP = 249–251 °C; ^1^H NMR (400 MHz, DMSO-*d*_6_) *δ* 10.66 (s, 2H), 8.67 (s, 2H), 8.31 (s, 2H), 7.85 (d, *J* = 8.6 Hz, 4H), 7.63 (d, *J* = 8.8 Hz, 4H), 7.41 (d, *J* = 8.8 Hz, 4H), 7.20 (d, *J* = 8.6 Hz, 4H), 5.39 (s, 4H), 5.28 (s, 4H); ^13^C NMR (101 MHz, DMSO-*d*_6_) *δ* 164.88, 161.02, 160.94, 142.62, 137.84, 130.47, 129.35, 127.84, 127.33, 126.97, 121.25, 115.61, 61.63, 52.68; anal. calcd: C_36_H_30_Cl_2_N_10_O_4_; C, 58.62; H, 4.10; N, 18.99; found C, 58.80; H, 4.27; N, 19.09.

#### 2,2′-((((((1*E*,1′*E*)-Hydrazine-1,2-diylidenebis(methaneylylidene))bis(4,1-phenylene))bis(oxy))bis(methylene))bis(1*H*-1,2,3-triazole-4,1-diyl))bis(*N*-(2,4-dichlorophenyl)acetamide) (10k)

Yellow solid; yield: 75%; MP = 252–254 °C; ^1^H NMR (400 MHz, DMSO-*d*_6_) *δ* 10.20 (s, 2H), 8.66 (s, 2H), 8.31 (s, 2H), 7.84 (d, *J* = 8.6 Hz, 4H), 7.82–7.77 (m, 3H), 7.73 (d, *J* = 2.1 Hz, 2H), 7.45 (dd, *J* = 8.7, 2.3 Hz, 2H), 7.23–7.16 (m, 3H), 5.49 (s, 4H), 5.28 (s, 4H); ^13^C NMR (101 MHz, DMSO-*d*_6_) *δ* 165.56, 161.03, 160.94, 142.64, 133.85, 130.47, 130.37, 129.58, 128.21, 127.60, 127.32, 127.01, 115.60, 105.02, 61.63, 52.41; anal. calcd: C_36_H_28_Cl_4_N_10_O_4_; C, 53.61; H, 3.50; N, 17.37; found C, 53.82; H, 3.68; N, 17.52.

#### 2,2′-((((((1*E*,1′*E*)-Hydrazine-1,2-diylidenebis(methaneylylidene))bis(4,1-phenylene))bis(oxy))bis(methylene))bis(1*H*-1,2,3-triazole-4,1-diyl))bis(*N*-(4-bromophenyl)acetamide) (10l)

Brown solid; yield: 89%; MP = 273–275 °C; ^1^H NMR (400 MHz, DMSO-*d*_6_) *δ* 10.66 (s, 2H), 8.67 (s, 2H), 8.31 (s, 2H), 7.85 (d, *J* = 8.6 Hz, 4H), 7.58 (d, *J* = 9.0 Hz, 4H), 7.54 (d, *J* = 9.0 Hz, 4H), 7.20 (d, *J* = 8.6 Hz, 4H), 5.39 (s, 4H), 5.28 (s, 4H); ^13^C NMR (101 MHz, DMSO-*d*_6_) *δ* 164.90, 161.03, 160.94, 142.62, 138.25, 132.26, 130.48, 127.32, 126.97, 121.63, 115.90, 115.60, 61.63, 52.70; anal. calcd: C_36_H_30_Br_2_N_10_O_4_; C, 52.32; H, 3.66; N, 16.95; found C, 52.41; H, 3.88; N, 17.17.

#### 2,2′-((((((1*E*,1′*E*)-Hydrazine-1,2-diylidenebis(methaneylylidene))bis(4,1-phenylene))bis(oxy))bis(methylene))bis(1*H*-1,2,3-triazole-4,1-diyl))bis(*N*-(2-nitrophenyl)acetamide) (10m)

Brown solid; yield: 77%; MP = 211–213 °C; ^1^H NMR (400 MHz, DMSO-*d*_6_) *δ* 10.88 (s, 2H), 8.69 (s, 2H), 8.39 (s, 2H), 8.10 (d, *J* = 8.0 Hz, 2H), 7.94 (d, *J* = 8.6 Hz, 4H), 7.84 (m, 4H), 7.54 (t, *J* = 7.1 Hz, 2H), 7.30 (d, *J* = 8.7 Hz, 4H), 5.57 (s, 4H), 5.38 (s, 4H); ^13^C NMR (101 MHz, DMSO-*d*_6_) *δ* 165.93, 161.11, 160.94, 142.95, 142.90, 134.86, 130.97, 130.97, 127.83, 126.96, 126.72, 126.34, 125.67, 115.80, 61.68, 52.46; anal. calcd: C_36_H_30_N_12_O_8_; C, 56.99; H, 3.99; N, 22.15; found C, 57.21; H, 4.15; N, 22.34.

#### 2,2′-((((((1*E*,1′*E*)-Hydrazine-1,2-diylidenebis(methaneylylidene))bis(4,1-phenylene))bis(oxy))bis(methylene))bis(1*H*-1,2,3-triazole-4,1-diyl))bis(*N*-(4-nitrophenyl)acetamide) (10n)

Brown solid; yield: 79%; MP = 232–234 °C; ^1^H NMR (400 MHz, DMSO-*d*_6_) *δ* 11.12 (s, 2H), 8.67 (s, 2H), 8.33 (s, 2H), 8.27 (d, *J* = 9.1 Hz, 4H), (7.87–7.83) (m, 8H), 7.20 (d, *J* = 8.6 Hz, 4H), 5.47 (s, 4H), 5.30 (s, 4H); ^13^C NMR (101 MHz, DMSO-*d*_6_) *δ* 165.84, 161.02, 160.94, 144.98, 143.09, 130.48, 127.34, 127.00, 125.64, 119.53, 115.62, 61.65, 52.82; anal. calcd: C_36_H_30_N_12_O_8_; C, 56.99; H, 3.99; N, 22.15; found C, 57.15; H, 4.14; N, 22.33.

### Anti-cholinesterase assay

The inhibition effects of the new 2,2′-(1*E*,1′*E*)-hydrazine-1,2-diylidenebis(phenoxy-1,2,3-triazole-*N*-phenylacetamide) derivatives 10a–n against AChE and BChE were determined by Ellman's spectrophotometric method according to previously described methods.^27^

### α-Glucosidase inhibition assay

The inhibitory activity of the new compounds 10a–n against α-glucosidase was evaluated according to the protocol described in the literature.^25^ For this assay, the yeast form of α-glucosidase was used and *p*-nitrophenyl-α-d-glycopyranoside (*p*-NPG) was used as the substrate. The required solutions of these materials were prepared in a phosphate buffer solution (pH of 7.4 and a concentration of 5 mM). Sample dilutions were prepared by dissolving 5 mg of the synthesized compounds 10a–n in 5 mL of ethanol/water. Subsequently, a mixture containing 200 μL of phosphate solution, 50 μL of the substrate, and 5–200 μL of the sample solution was pre-incubated at 35 °C for 3 min for α-glucosidase. The absorbance for the α-glucosidase inhibition assay was measured at 405 nm to determine the inhibitory activities of the synthesized compounds.

### Molecular docking study

Docking studies of the most potent compounds were performed on AChE, BChE, and α-glucosidase according to our previously reported works.^28,29^ In this regard, compound 10k (as the most potent compound against AChE and BChE) and compound 10h (as the most potent against α-glucosidase) were placed in the active site of the target enzymes AChE (pdb code: 1EVE), BChE (pdb code: 1P0I), and modeled α-glucosidase by AutoDock Tools 1.5.6. The 3D structures of the compounds 10k and 10h were constructed by MarvinSketch 5.10.4, and the pdbqt formats of these compounds were prepared by AutoDock Tools. The pdbqt structures of the target enzymes were also prepared using AutoDock software. The grid box size for AChE was set at 40 × 40 × 40 Å, and the center of this box was set at *x* = 2.023, *y* = 63.295, and *z* = 67.062 Å. Moreover, the grid box size for BChE was set at 56 × 56 × 56 Å, and the center of it was set at *x* = 137.985, *y* = 122.725, and *z* = 38.78 Å. The parameters for α-glucosidase were set as follows: grid box size: 40 × 40 × 40 Å and the center of this box: *x* = 12.5825, *y* = −7.8955, and *z* = 12.519 Å. 50 runs were used for each docked system by AutoDock search (by the Lamarckian genetic algorithm). Finally, the best pose of the selected compounds 10k and 10h was analyzed by Discovery Studio 2019 Client (Accelrys, Inc., San Diego, CA).

### Cell culture

The human neuroblastoma cell line SH-SY5Y (ATCC® CRL-2266™) and HEK293 (ATCC® CRL-1573™) human embryonic kidney cells were grown in Dulbecco's Modified Eagle Medium/Nutrient Mixture F-12 (DMEM/F-12) culture containing 10% heat-inactivated fetal bovine serum (FBS) and 1% antibiotics. The cells were incubated at 37 °C in a humidified atmosphere of 5% CO_2_. Both cell lines were provided by the American Type Culture Collection (ATCC).^30,31^

### Cell viability assay

SH-SY5Y (ATCC® CRL-2266™) and HEK293 (ATCC® CRL-1573™) cells were seeded into 96-well plates at a density of 5 × 10^3^ cells per well, and allowed to adhere overnight in a humidified incubator at 37 °C with 5% CO_2_. After attachment, the cells were treated with eight different concentrations of the target compounds (200, 100, 50, 25, 12.5, 6.25, 3.125, and 1.5625 μM), prepared *via* serial 1 : 2 dilutions, for 24 hours. Each concentration was tested in triplicate.

Following treatment, 10 μL of MTT solution (5 mg mL^−1^ in PBS) was added to each well and incubated for an additional 4 hours under the same conditions. At the end of the incubation period, the medium containing MTT was carefully removed, and the formazan crystals formed by viable cells were solubilized in 100 μL of DMSO per well. The absorbance was measured at 570 nm using a microplate reader. The net absorbance (after blank subtraction) was used to calculate the percentage cell viability relative to the untreated control cells, which was set at 100%. All results were expressed as mean ± standard deviation (SD) of three independent experiments.^32,33^

### Prediction of water solubility and intestinal absorption

Two of the most potent compounds 10h and 10k were evaluated *in silico* in terms of water solubility and intestinal absorption by pkCSM online software.^34^

## Results and discussion

### Chemistry

**Scheme 1:**
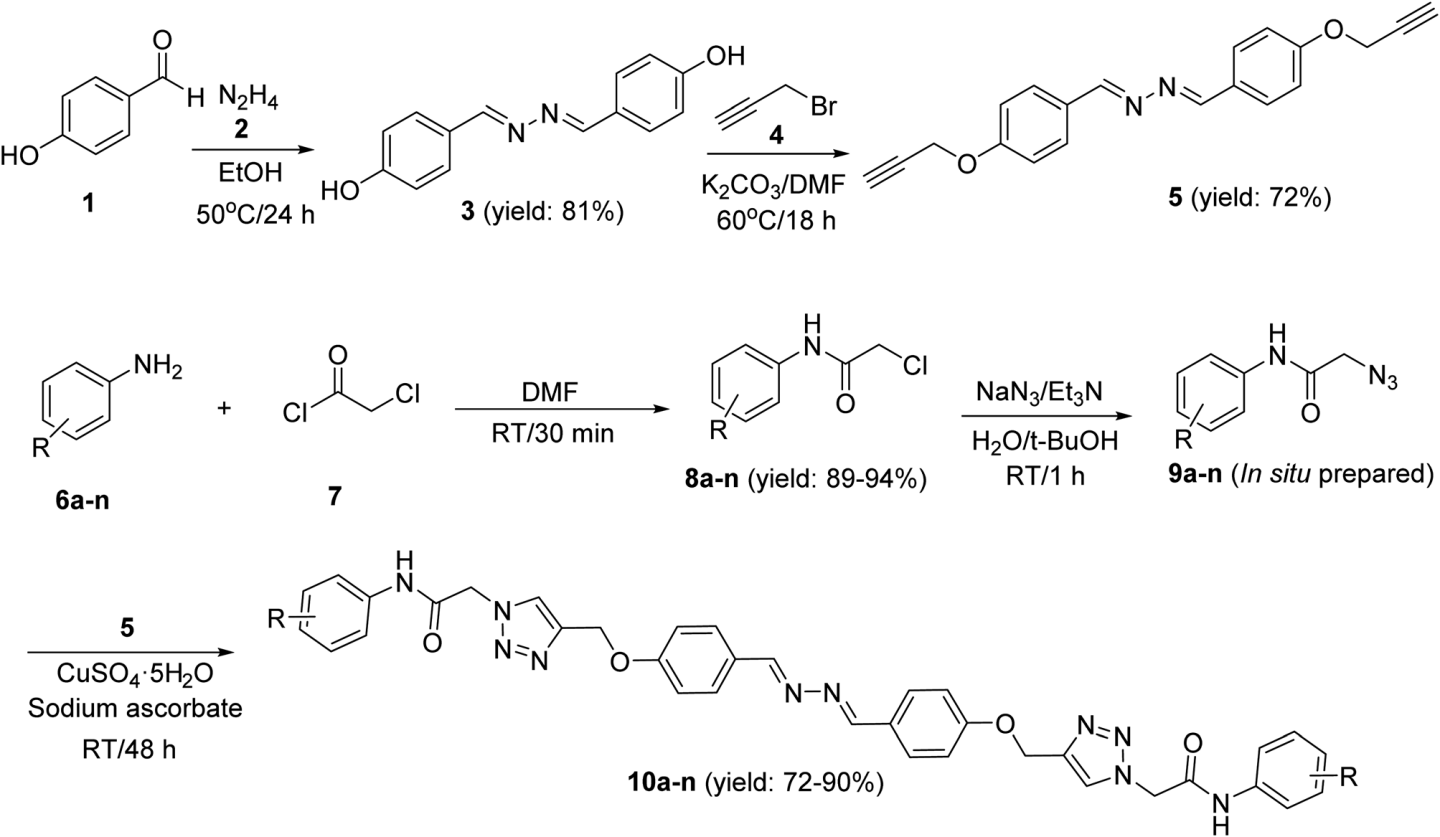
Synthesis of 2,2′-((1*E*,1′*E*)-hydrazine-1,2-diylidenebis(phenoxy-1,2,3-triazole-*N*-phenylacetamide) derivatives 10a–n.

New 2,2′-(1*E*,1′*E*)-hydrazine-1,2-diylidenebis(phenoxy-1,2,3-triazole-*N*-phenylacetamide) derivatives 10a–n were synthesized according to the depicted procedure in Scheme 1. According to the click reaction, the propargylated structure required to produce these 1,2,3-triazole derivatives is 1,2-bis((*E*)-4-(prop-2-yn-1-yloxy)benzylidene)hydrazine (5). The latter compound was obtained by the following reactions: (1) reaction between 4-hydroxybenzaldehyde (1) and hydrazine (2), which led to the formation of 4,4′-((1*E*,1′*E*)-hydrazine-1,2-diylidenebis(methaneylylidene))diphenol (3); and (2) reaction of the compound 3 and propargyl bromide (4), which led to the formation of the propargylated derivative 5. According to the click reaction, the other structures required for the formation of our target compounds are azide derivatives 9a–n, which were obtained by our previously reported method.^25^ Finally, the reaction between compound 5 and azide derivatives 9a–n led to the formation of new 2,2′-(1*E*,1′*E*)-hydrazine-1,2-diylidenebis(phenoxy-1,2,3-triazole-*N*-phenylacetamide) derivatives 10a–n.

### Enzymatic inhibition assays

All of the new synthesized compounds 10a–n were evaluated against three important metabolic enzymes AChE, BChE, and α-glucosidase, and the obtained data were compared to the appropriate positive controls.

In terms of the anti-ChE activity, our results demonstrated that all of the compounds 10a–n were more potent than the positive control tacrine against BChE. Furthermore, most of these compounds were more potent than this control against AChE. On the other hand, comparison of the IC_50_ values of the positive control donepezil against AChE and BChE with the observed IC_50_ values for compounds 10a–n demonstrated that all the compounds 10a–n were more potent than donepezil against BChE, but the latter compounds were less potent than donepezil against BChE. In terms of the anti-α-glucosidase activity, most of the new compounds were more potent than the positive control acarbose.

**Fig. 3:**
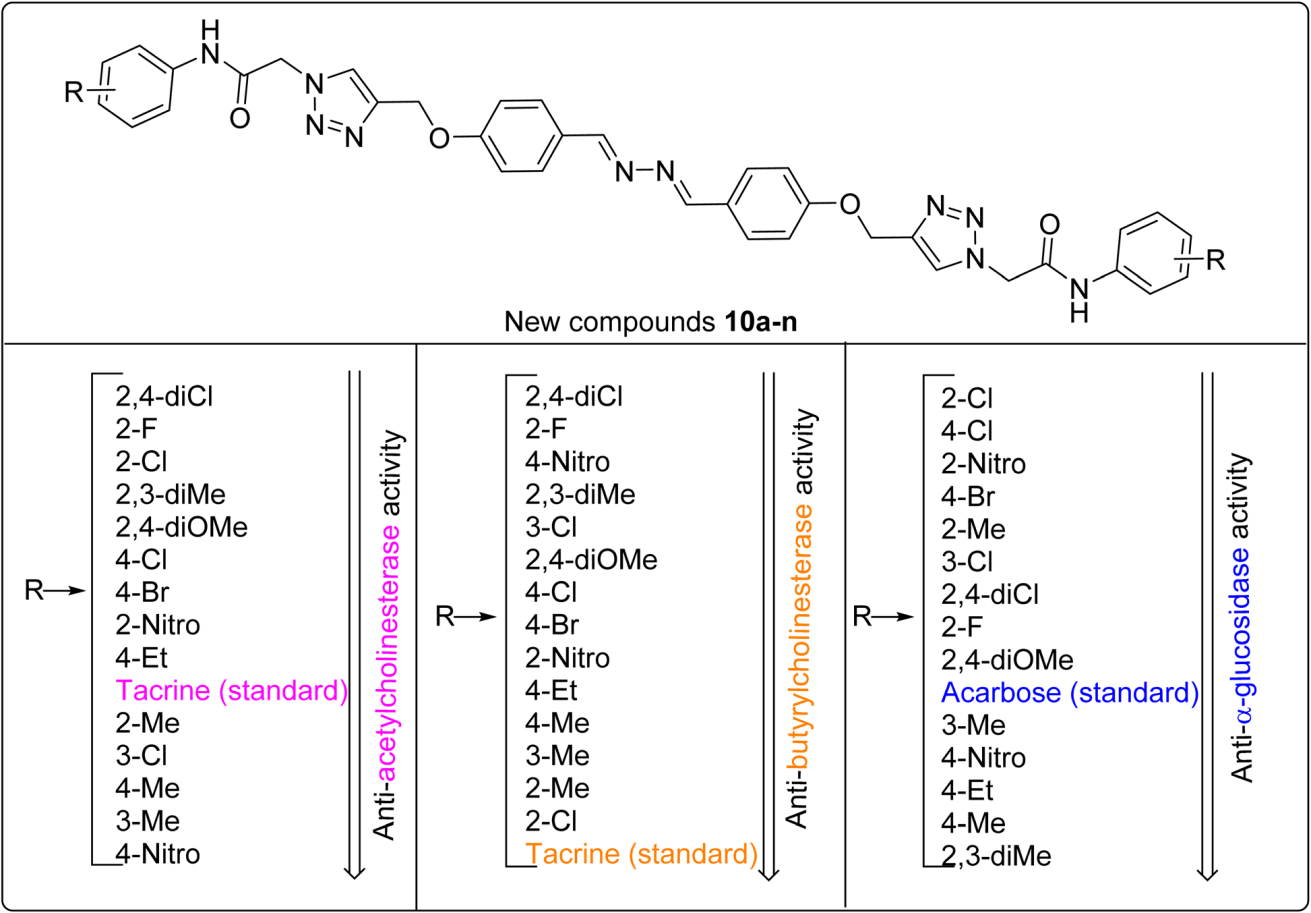
SAR diagrams of compounds 10a–n in anti-AChE, anti-BChE, and anti-α-glucosidase assays.

As can be seen in Scheme 1 and Table 1, the title compounds 10a–n differ based on the substituents on the terminal phenyls. The structure–activity relationships (SAR) of the new compounds in anti-AChE, anti-BChE, and anti-α-glucosidase assays are schematically depicted in Fig. 3.

**Table 1:** Enzyme inhibition results of novel compounds 10a–n against AChE, BChE and alpha glucosidase

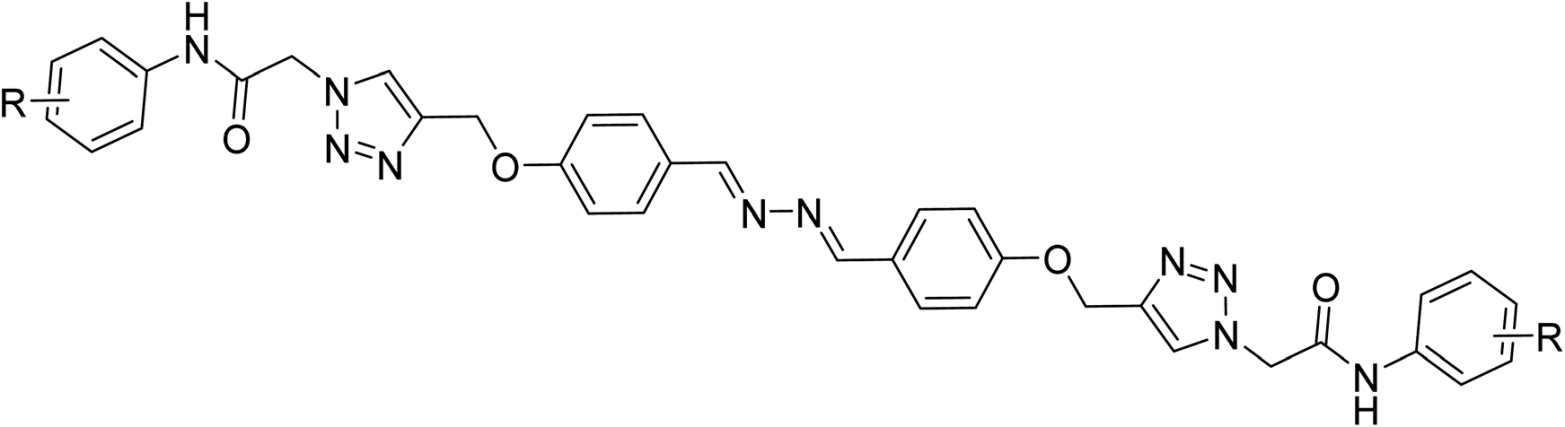				
Compound	R	IC_50_ (nM)		
		AChE	BChE	α-Glucosidase
10a	2-Me	163.30 ± 5.38	18.43 ± 3.09	43.09 ± 8.21
10b	3-Me	166.80 ± 9.46	18.28 ± 4.84	62.09 ± 5.43
10c	4-Me	164.23 ± 9.70	17.12 ± 3.21	66.98 ± 7.88
10d	2,3-Dimethyl	131.01 ± 7.23	14.95 ± 2.08	71.19 ± 8.14
10e	2,4-Dimethoxy	139.76 ± 9.43	16.28 ± 2.09	58.45 ± 4.27
10f	4-Et	159.43 ± 6.17	17.02 ± 5.46	66.39 ± 4.43
10g	2-F	112.23 ± 9.60	12.43 ± 3.21	56.66 ± 6.34
10h	2-Cl	118.43 ± 9.17	19.43 ± 0.65	23.90 ± 3.42
10i	3-Cl	163.74 ± 9.54	15.32 ± 3.16	45.84 ± 4.71
10j	4-Cl	145.08 ± 6.23	16.43 ± 4.43	34.32 ± 3.49
10k	2,4-Dichloro	105.24 ± 7.32	10.11 ± 1.08	46.12 ± 3.26
10l	4-Br	148.19 ± 8.23	16.45 ± 4.74	39.13 ± 5.10
10m	2-Nitro	156.76 ± 8.65	16.91 ± 4.83	36.62 ± 4.86
10n	4-Nitro	172.91 ± 9.71	13.90 ± 4.30	65.09 ± 6.01
Tacrine	—	160.87 ± 6.20	58.03 ± 9.80	—
Donepezil	—	32.87 ± 2.16	>1000	—
Acarbose	—	—	—	60.57 ± 5.78

As can be seen in Table 1 and Fig. 3, in the anti-AChE assay, the most potent compound was 2,4-dichloro derivative 10k. Removing the 2-chloro substituent, as in the case of the 4-chloro derivative 10j, diminished the inhibitory activity to 1.3-fold. The second most potent compound against AChE was 2-fluoro derivative 10g. Replacement of this substituent with a chlorine atom created a negligible decrease in the anti-AChE activity, as observed in compound 10h as the third most potent compound. Among our new synthesized compounds, the fourth and fifth most potent compounds were the 2,3-dimethyl derivative 10d and 2,4-dimethoxy derivative 10e. The inhibitory activities of compounds with the substituents 4-Cl, 4-Br, 2-NO_2_, and 4-ethyl were negligibly better than tacrine (positive control), and compounds with substituents 2-methyl, 3-Cl, 4-methyl, 3-methyl, and 4-NO_2_ were less potent than tacrine against AChE.

In term of the inhibitory activity of new compounds 10a–n against BChE, all new compounds were more potent than tacrine. The most potent compound was the 2,4-dichloro derivative 10k with an IC_50_ value of 10.53, and the least potent compound was 2-chloro derivative 10h with an IC_50_ value of 19.43 nM (Table 1 and Fig. 3). These compounds were 5.5- and 2.9-folds more potent than tacrine (IC_50_ value = 58.03 nM), respectively. The remaining derivatives had IC_50_ values ranging from 12.43 to 18.43 nM.

Among all of the synthesized compound 10a–n, nine derivatives were more potent than the positive control acarbose against α-glucosidase. As can be seen in Table 1 and Fig. 3, the most potent compounds were 2-chloro derivative 10h and 4-chloro derivative 10j. These compounds, respectively, were 2.7- and 1.7-folds more potent than the standard inhibitor acarbose. The order of anti-α-glucosidase activity in the 2-substituented compounds was Cl > NO_2_ > Me > F. In the 4-substituented compounds, the order was Cl > Br > NO_2_ > Me (Fig. 3). Moreover, the chlorine atom in the 3-position also acted better than the methyl group in this position. In contrast, 2,4-dichloro derivative 10k was a moderate inhibitor in comparison to the other chloro derivatives.

### Kinetic study

**Fig. 4:**
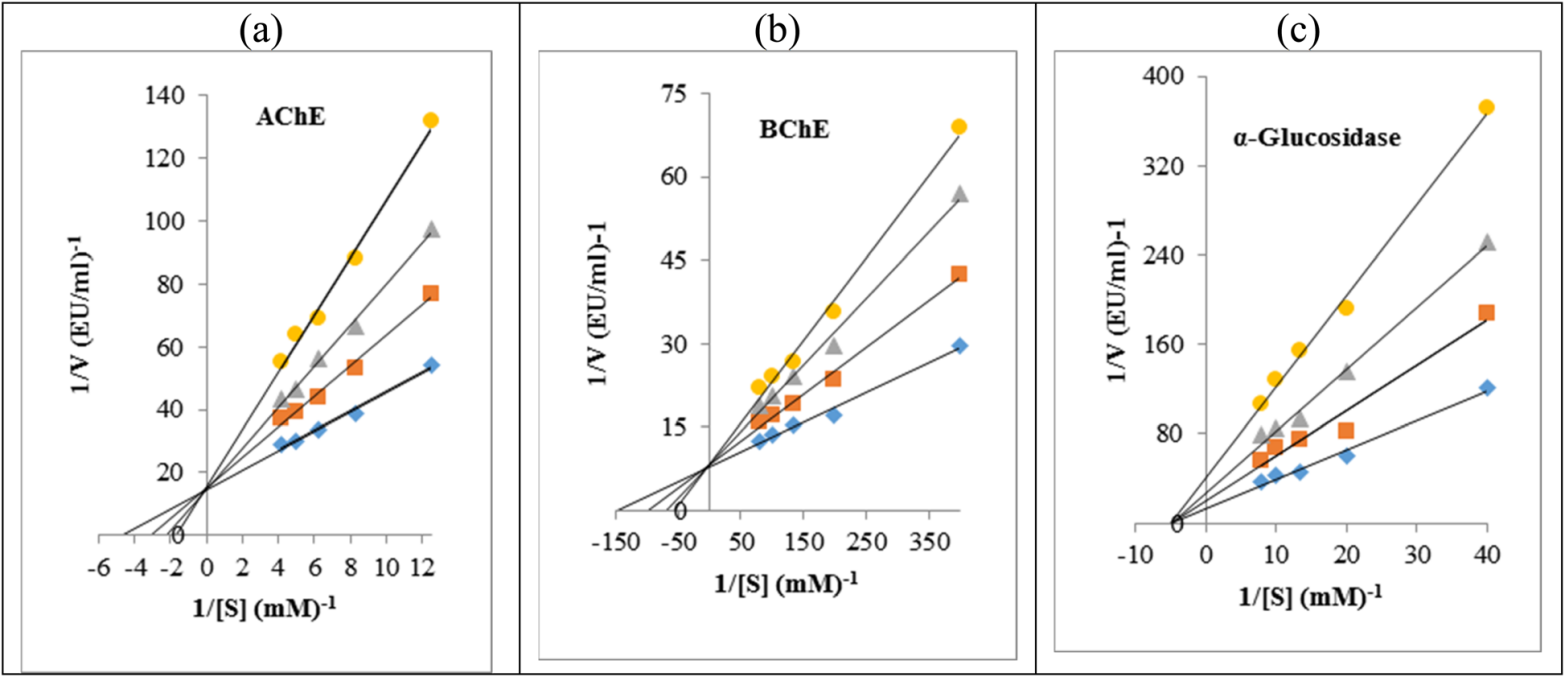
Kinetic study of compound 10k against AChE (a) and BChE (b), and of compound 10h against α-glucosidase (c).

To evaluate the inhibition mechanism of the new synthesized compounds, kinetics studies were performed on the most potent compounds against AChE, BChE, and α-glucosidase. In this regard, compound 10k for AChE and BChE and compound 10h for α-glucosidase were selected. The type of inhibition of these compounds was determined by Lineweaver–Burk plots. As can be seen in Fig. 4a and b, the kinetic study against AChE and BChE demonstrated that in the presence of compound 10k, the values of *V*_max_ remained unchanged while the values of *K*_m_ increased. These indicated a competitive type of inhibition for compound 10k. In contrast, the Lineweaver–Burk plots of compound 10h against α-glucosidase showed that with increasing concentrations of this compound, the *V*_max_ value decreased while the *K*_m_ value remained unchanged. This behavior demonstrated that compound 10h is a non-competitive inhibitor against α-glucosidase.

### Docking study

To evaluate the interaction mode of the new synthesized compounds in the active sites of AChE, BChE, and α-glucosidase as target enzymes in the presented work, a docking study was performed on the most potent compounds against these enzymes.

As mentioned in the introduction, there are four important components at the active sites of AChE and BChE.^12^ These components, their sub-components and the available amino acids in these sub-components are listed in Table 2.

**Table 2:** Details of the important components of the active sites of AChE and BChE

Component	Sub-component	Available amino acids	
		AChE	BChE
CAS	AS	Trp84, Phe331, Phe330	Trp82, Tyr128, Phe329
	CT	Ser200, His440, Glu327	Ser198, His438, Glu325
PAS	—	Asp72, Tyr334, Tyr121, Trp279, Tyr70	Asp70, Tyr332
Acyl pocket	—	Phe290, Phe228	Val288, Leu286
Oxyanion hole	—	Ala201, Gly118, Gly119	Ala199, Gly116, Gly117

**Fig. 5:**
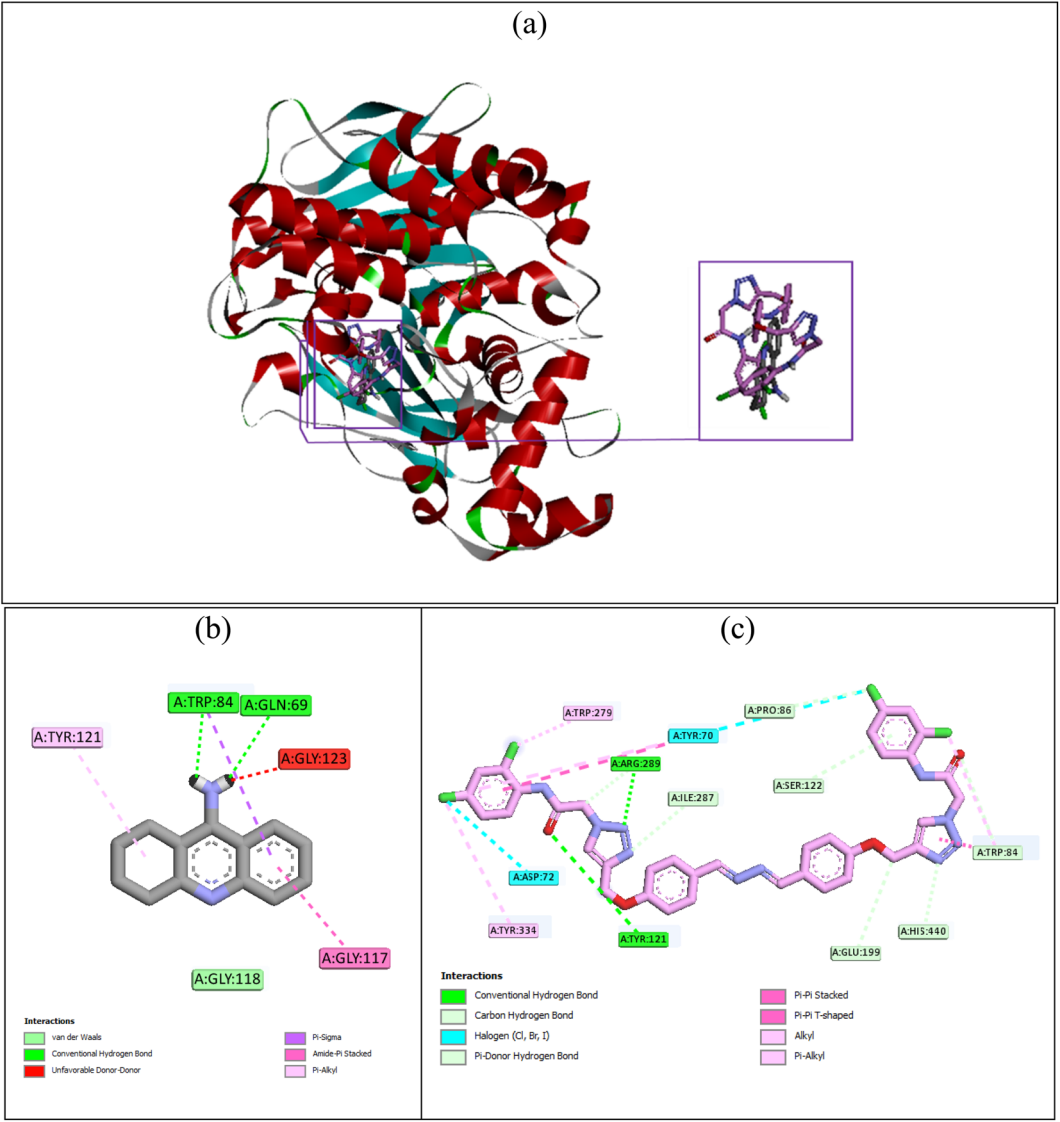
Superimposed structure of tacrine (gray) and compound 10k (pink) in the AChE's active site (a). Interaction modes of tacrine (b) and compound 10k (c) in the AChE's active site.

For the first step, tacrine and the most potent 10k were placed in the AChE's active site. The superimposed structure of these compounds is depicted in Fig. 5a. The interaction mode of the standard inhibitor tacrine is shown in Fig. 5b. The latter figure showed that tacrine established two hydrogen bonds with Trp84 (AS) and Gln69, a van der Waals interaction with Gly118 (OAH), and an unfavorable interaction with Gly123. Furthermore, this inhibitor formed three hydrophobic interactions with residues Tyr121 (PAS), Gly117, and Trp84 (AS). The binding energy of tacrine in the AChE's active site was −7.68 kcal mol^−1^. Our most potent new compound 10k with the binding energy value of −13.5 kcal mol^−1^ established the following important interactions in the active site of AChE: two hydrogen binds with residues Tyr121 (PAS) and Arg289, and two halogen atom-mediated interactions with residues Asp72 and Tyr70 (PAS) (Fig. 5c). Compound 10k also formed several non-classical hydrogen bonds with residues Ile287, Pro86, Ser122, Trp84 (AS), His440, and Glu199, and several hydrophobic interactions with residues Tyr334, Trp279, Tyr70 (PAS), and Trp84 (AS).

**Fig. 6:**
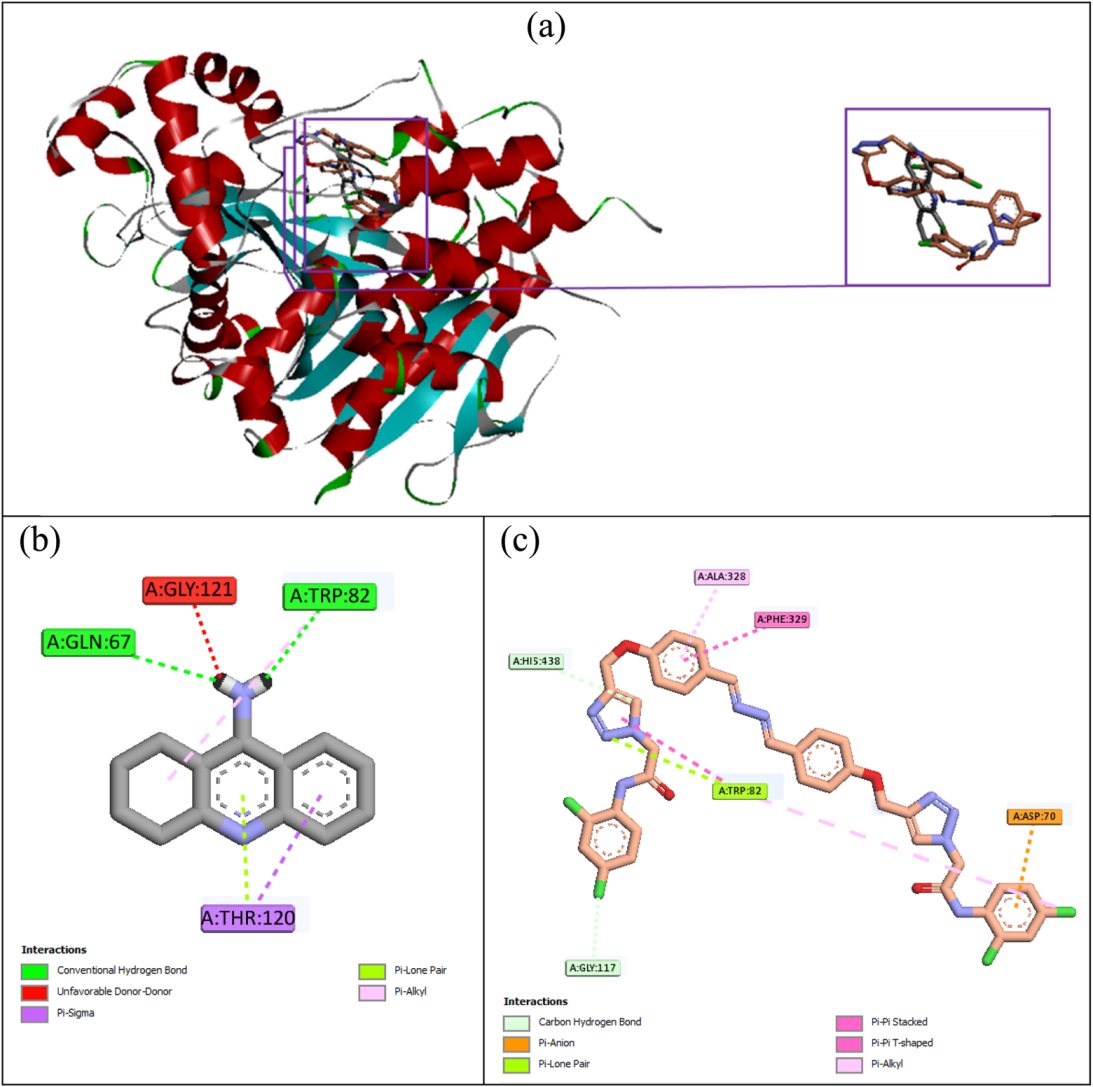
Superimposed structure of tacrine (gray) and compound 10k (orange) in the BChE's active site (a). Interaction modes of tacrine (b) and compound 10k (c) in the BChE's active site.

Compound 10k also was the most potent inhibitor against BChE. The superimposed structure of this compound and the standard inhibitor tacrine in the BChE's active site is shown in Fig. 6a. Tacrine with the binding energy value of −6.9 kcal mol^−1^ established two hydrogen bonds with Gln67 and Trp82 (AS). The latter amino acid also formed a hydrophobic interaction with tacrine. Furthermore, between tacrine and BChE's active site, an unfavorable interaction with the residue Gly121 and a π-lone pair, and π-sigma interactions with residue Thr120 were also observed (Fig. 6b). Compound 10k, as the best compound against BChE, established the following π-interactions with the active site of this enzyme: a π-anion interaction with Asp70 (PAS), two π–π interactions with Phe329 (AS) and Trp82 (AS), and a π-lone pair interaction with Trp82 (AS). Furthermore, this compound formed a non-classical hydrogen bond with His438 (AS) and two hydrophobic interactions with Ala328 and Trp82 (AS). The binding energy of compound 10k was −10.45 kcal mol^−1^.

### Cytotoxicity studies

In order to evaluate the potential cytotoxic effects of the synthesized compounds, *in vitro* cell viability assays were conducted on two different human cell lines: SH-SY5Y as a neuroblastoma cell line and HEK293 embryonic kidney cells as a normal cell line. The SH-SY5Y cell line is widely used as a neuronal model due to its capacity for differentiation into neuron-like cells and its relevance to neurodegenerative disorders such as Alzheimer's disease.^35^ As such, it serves as a valuable tool for screening the neuroprotective or neurotoxic properties of novel compounds.^36^ In parallel, HEK293 cells were employed as a non-neuronal, non-cancerous control to assess the general cytotoxicity and cellular selectivity. The cytotoxicity profiles of compounds 10a–n against both SH-SY5Y and HEK293 cell lines were assayed by MTT method, and the obtained results are summarized in Table 3.

**Table 3:** Cell viability of SH-SY5Y and HEK293 cells treated with the compounds for 24 h. Data are mean ± SD (*n* = 3)

Compounds	IC_50_ (μM)		
	SH-SY5Y	HEK-293	Selectivity
10a	8.22 ± 0.12	78.21 ± 0.42	9.5
10b	15.82 ± 0.20	43.42 ± 0.36	2.7
10c	11.93 ± 0.20	46.91 ± 0.32	3.9
10d	16.37 ± 0.21	75.22 ± 0.35	4.6
10e	33.19 ± 0.25	68.95 ± 0.41	2.1
10f	7.33 ± 0.11	33.18 ± 0.55	4.5
10g	12.94 ± 0.13	86.45 ± 0.52	6.7
10h	7.42 ± 0.07	78.38 ± 0.57	10.6
10i	4.26 ± 0.08	37.39 ± 0.55	8.8
10j	6.75 ± 0.18	67.43 ± 0.47	10.0
10k	8.11 ± 0.07	81.71 ± 0.56	10.1
10l	42.15 ± 0.37	92.86 ± 0.71	2.2
10m	28.33 ± 0.26	46.45 ± 0.45	1.6
10n	5.22 ± 0.12	38.42 ± 0.19	7.4
Sorafenib	3.88 ± 0.12	16.22 ± 0.14	4.2

The results indicate that several compounds exhibited substantial cytotoxic activity against SH-SY5Y cells, while displaying considerably lower toxicity toward HEK293 cells, suggesting potential selectivity toward cancerous cells. Among the tested molecules, compounds 10i (3-Cl), 10k (2,4-dichloro), 10h (2-Cl), 10a (2-Me), and 10j (4-Cl) stood out due to their strong cytotoxic effects on SH-SY5Y cells (IC_50_ values ranging from 4.26 to 8.22 μM), combined with high selectivity indices of 8.8 to 10.6. Notably, compound 10k exhibited an IC_50_ of 8.11 μM against SH-SY5Y and 81.71 μM against HEK293, corresponding to a selectivity index of 10.1, highlighting it as one of the most promising derivatives in terms of therapeutic selectivity. Such high selectivity suggests that the compound preferentially targets cancer cells over normal cells, a highly desirable trait in anticancer drug development. Several other synthesized derivatives showed moderate to low cytotoxicity and selectivity profiles. For instance, compound 10n (4-NO_2_) exhibited a relatively favorable activity with an IC_50_ of 5.22 μM and a selectivity index of 7.4, indicating that nitro substitution at the *para* position may contribute positively to anticancer effects. Similarly, compound 10g (2-F) showed moderate potency (IC_50_ = 12.94 μM) with a relatively high selectivity (SI = 6.7), while 10c (4-Me) and 10d (2,3-dimethyl) demonstrated moderate cytotoxic effects (IC_50_ between 12 and 16 μM) with modest selectivity indices ranging from 3.9 to 4.6. In contrast, compounds such as 10e (2,4-dimethoxy) and 10l (4-Br) displayed weak cytotoxicity against SH-SY5Y cells (IC_50_ > 30 μM) along with low selectivity, suggesting their limited potential as anticancer agents. Similarly, compound 10m (2-NO_2_) showed only modest activity (IC_50_ = 28.33 μM) and low selectivity (SI = 1.6), indicating that nitro substitution at the *ortho* position may not be favorable for cytotoxic effects. The reference compound sorafenib demonstrated strong potency (IC_50_ = 3.88 μM) but had a moderate selectivity index of 4.2, which was outperformed by several of the synthesized derivatives, including 10k, 10h, 10j, 10i, 10a, and 10n.

Overall, these results indicate that halogenated and methyl-substituted compounds show the most promising cytotoxicity and selectivity against SH-SY5Y neuroblastoma cells. Importantly, these compounds exhibit potent nanomolar enzyme inhibition without cytotoxicity at those active concentrations, especially in HEK293 cells, suggesting a favorable therapeutic window. Their selective toxicity toward neuroblastoma cells further supports their potential as targeted anticancer agents.

### Prediction of water solubility and intestinal absorption

Compounds 10k and 10h as two potent new compounds in enzymatic assays were studied in terms of two important pharmacokinetic properties, including water solubility and intestinal absorption (human), by pkCSM which is an online software.^34^ Our *in silico* studies predicted that the water solubility values of compounds 10h and 10k were −3.522 and −3.522 log mol L^−1^, respectively. These values are within an acceptable range for this property.^37^ On the other hand, it was predicted that the intestinal absorption (human) for compounds 10h and 10k are 89.235% and 91.589%, respectively. These values showed that our new compounds properly have high intestinal absorption.^34^

## Conclusion

ChE enzymes terminated ACh activity in nervous terminals, and inhibiting Ach activity has a special importance in the treatment of AD. Based on the reported structures for ChE inhibitors, in this work, new 1*E*,1′*E*-hydrazine-bis(phenoxy-1,2,3-triazol-acetamide) derivatives 10a–n were designed and synthesized as ChE inhibitors, and assigned against two important forms of ChE (AChE and BChE). Our new synthesized compounds have the necessary structural features to be α-glucosidase inhibitors. Therefore, all compounds 10a–n were also evaluated against α-glucosidase. *In vitro* ChE activity assay demonstrated that all title compounds were more potent than standard inhibitors tacrine and donepezil against BChE. With the exception of compounds 10a–c, 10i, and 10n, the remaining derivatives were more potent than tacrine against AChE. The anti-α-glucosidase assay showed that all new compounds, with the exception of compounds 10b–d, 10f, and 10n, were more potent than the standard inhibitor acarbose. The most potent compound against AChE and BChE was 2,4-dichloro derivative 10k, and the most potent compound against α-glucosidase was 2-chloro derivative 10h. Cell studies on new compounds 10a–n demonstrate that several derivatives, such as compounds 10a, 10h, 10j, and 10k, demonstrated notable cytotoxicity against neuroblastoma cells SH-SY5Y and low cytotoxicity on normal cells HEK293. Their pronounced cytotoxicity toward SH-SY5Y cells, coupled with minimal toxicity in non-cancerous cells, suggests their potential as selective anticancer agents for further preclinical development. Furthermore, according to docking studies, compounds 10h and 10k with favorable binding energies were attached to the active sites of the related target enzymes.

## Author contributions

MM and PT conceived, designed, and supervised this study. SK, FSKM, ND, AA, AD, and FM synthesized and interpreted the analytical data of the compounds. *In vitro* experiments were performed by HŞ, NS, and PT. *In silico* studies were performed and written by MM-K. The manuscript was drafted by MM-K, BL, and SS. MM reviewed and edited the draft.

## Conflicts of interest

The authors declare no conflict of interest.

## Supplementary Material

RA-015-D5RA03877D-s001

## Data Availability

The data supporting this article have been included as part of the SI. See DOI: https://doi.org/10.1039/d5ra03877d.
