# Supplementary material for: New 1E,1′E-hydrazine-bis(phenoxy-1,2,3-triazol-acetamide) derivatives as potent inhibitors against acetylcholinesterase, butyrylcholinesterase, and α-glucosidase
Source: RSC Adv. 2025 Aug 22;15(36):29960–71. doi: 10.1039/d5ra03877d (PMC12377313; doi:10.1039/d5ra03877d)
Supplement: RA-015-D5RA03877D-s001 [file RA-015-D5RA03877D-s001.pdf]

Supplementary Information (SI) for RSC Advances.
This journal is © The Royal Society of Chemistry 2025

Support information

New 1E,1'E-hydrazine-bis(phenoxy-1,2,3-triazol-acetamide) derivatives as potent inhibitors against acetylcholinesterase, butyrylcholinesterase, and α-glucosidase

Shahab Kermaninia[1]†, Maryam Mohammadi-Khanaposhtani[2]†, Halil Şenol[3], Fatemeh Sadat Khajeh Mohammadilar[1], Navid Dastyafteh[4], Fatemeh Moradkhani[1], Saeedeh Saeedi[1], Bagher Larijani[1], Armin Dadgar[5], Aydın Aktaş[6], Nastaran Sadeghian[7], Parham Taslimi[7]*, Mohammad Mahdavi[1]*

[1] Endocrinology and Metabolism Research Center, Endocrinology and Metabolism Clinical Sciences Institute, Tehran University of Medical Sciences, Tehran, Iran

[2] Cellular and Molecular Biology Research Center, Health Research Institute, Babol University of Medical Sciences, Babol, Iran.

[3] Department of Pharmaceutical Chemistry, Faculty of Pharmacy, Bezmialem Vakif University, 34093, Fatih, Istanbul, Türkiye.

[4] Pharmaceutical and Heterocyclic Compounds Research Laboratory, Department of Chemistry, Iran University of Science and Technology, Tehran 16846-13114, Iran.

[5] Drug Design and Development Research Center, The Institute of Pharmaceutical Sciences (TIPS), Tehran University of Medical Sciences, Tehran, Iran

[6] Vocational School of Health Service, Inonu University, Malatya, Türkiye

[7] Department of Biotechnology, Faculty of Science, Bartin University, Bartin, Türkiye

† These authors contributed equally to this work.

*Corresponding authors.

E-mail addresses: parham_taslimi_un@yahoo.com (P. Taslimi), momahdavi@tums.ac.ir (M. Mahdavi).

2,2'-((((((1E,1'E)-hydrazine-1,2-diylidenebis(methaneylylidene))bis(4,1-phenylene))bis(oxy))bis(methylene))bis(1H-1,2,3-triazole-4,1-diyl))bis(N-(o-tolyl)acetamide) (10a)

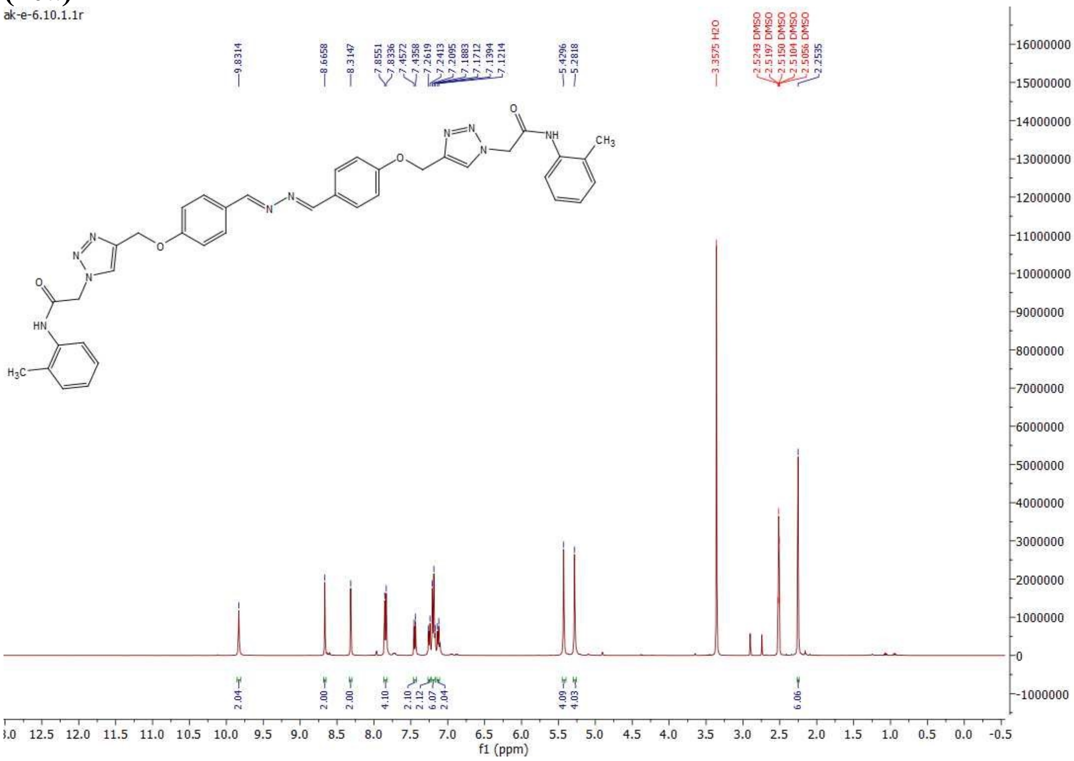

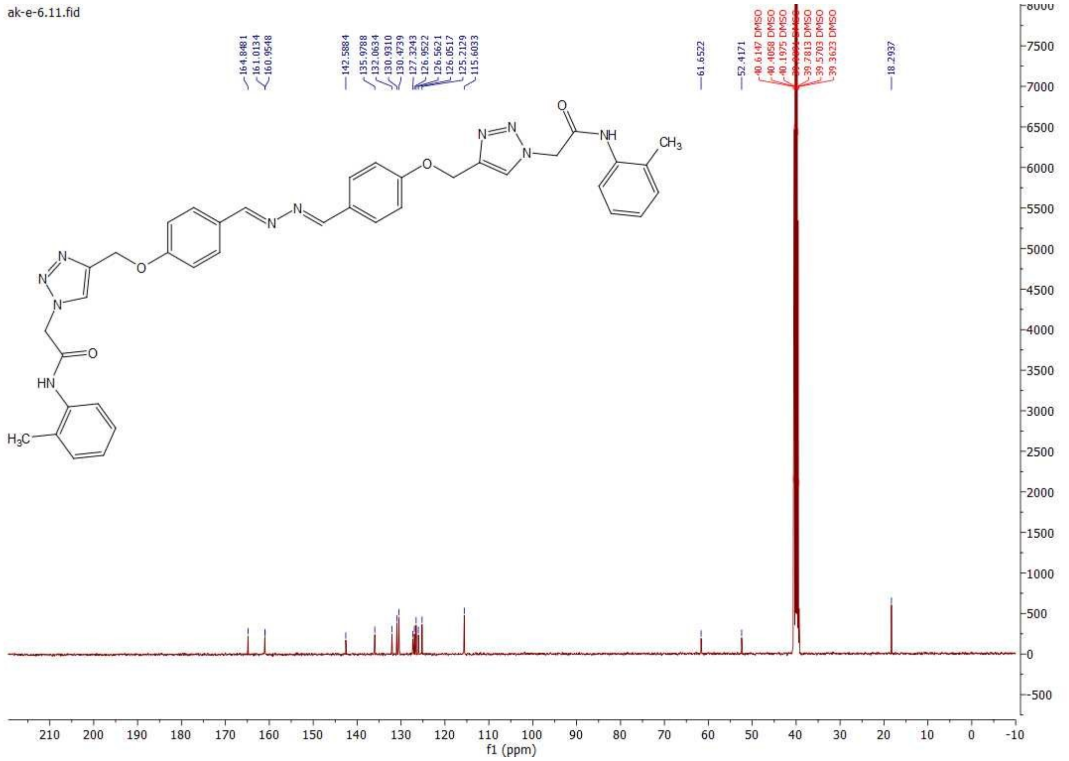

2,2'-((((((1E,1'E)-hydrazine-1,2-diylidenebis(methaneylylidene))bis(4,1-phenylene))bis(oxy))bis(methylene))bis(1H-1,2,3-triazole-4,1-diyl))bis(N-(m-tolyl)acetamide) (10b)

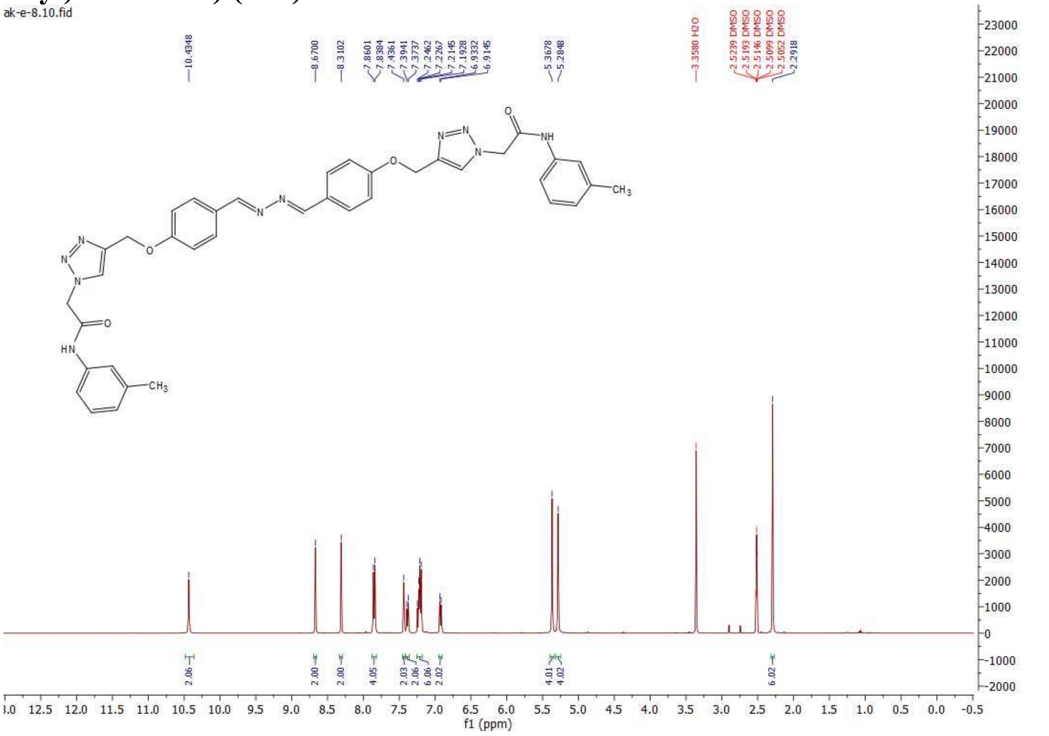

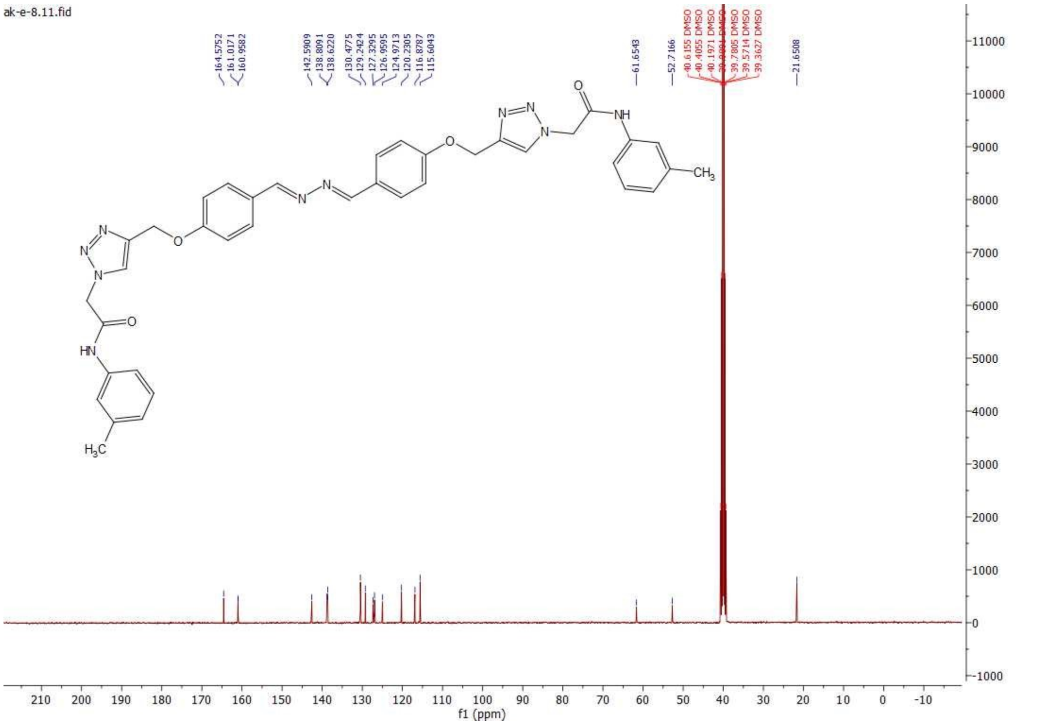

2,2'-((((((1E,1'E)-hydrazine-1,2-diylidenebis(methaneylylidene))bis(4,1-phenylene))bis(oxy))bis(methylene))bis(1H-1,2,3-triazole-4,1-diyl))bis(N-(p-tolyl)acetamide) (10c)

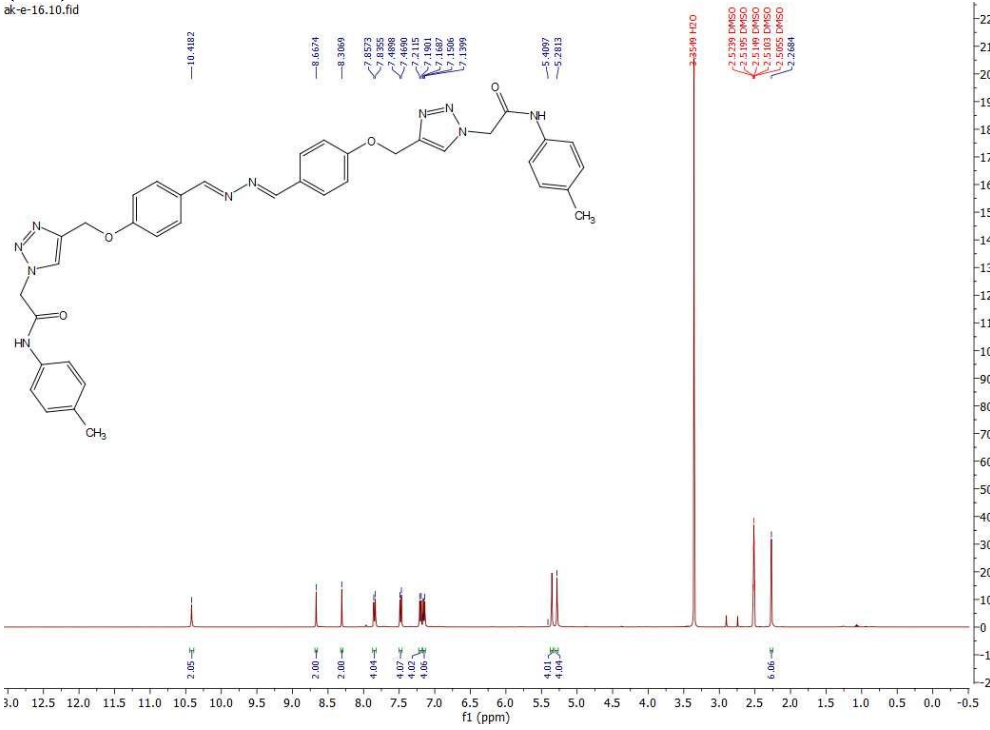

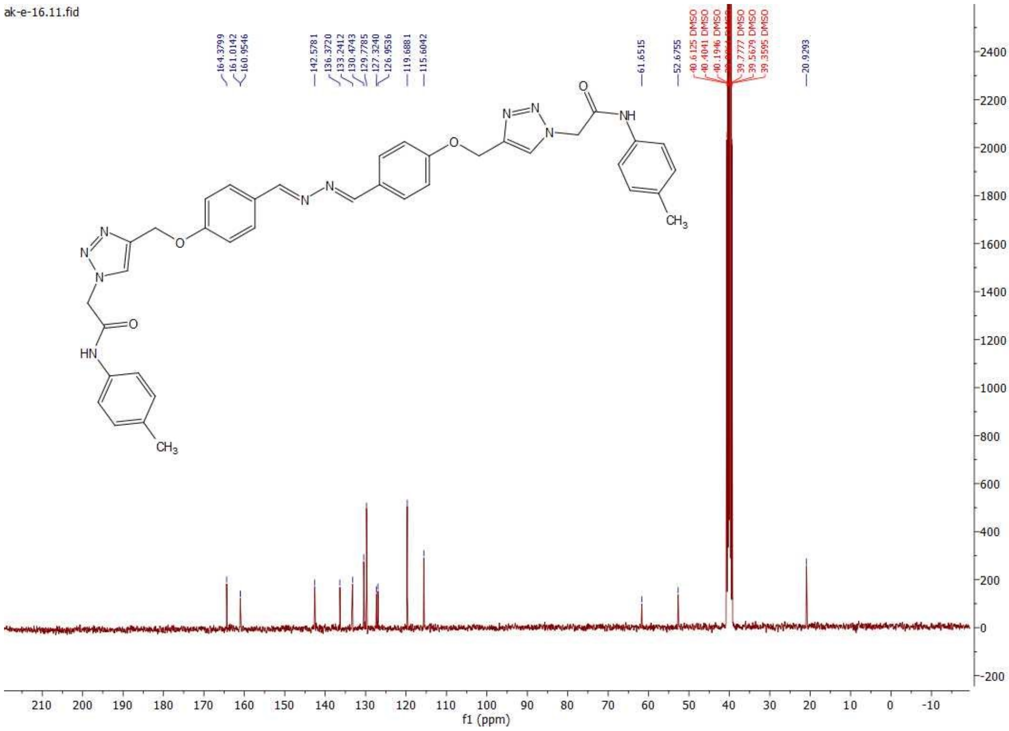

2,2'-((((((1E,1'E)-hydrazine-1,2-diylidenebis(methaneylylidene))bis(4,1-phenylene))bis(oxy))bis(methylene))bis(1H-1,2,3-triazole-4,1-diyl))bis(N-(2,3-dimethylphenyl)acetamide) (10d)

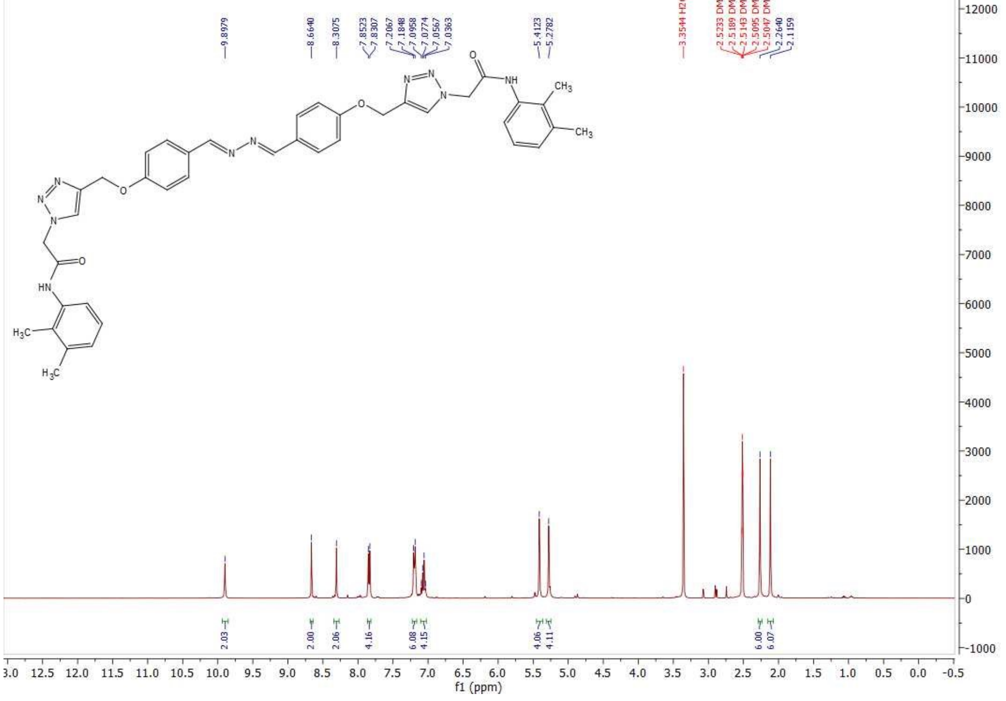

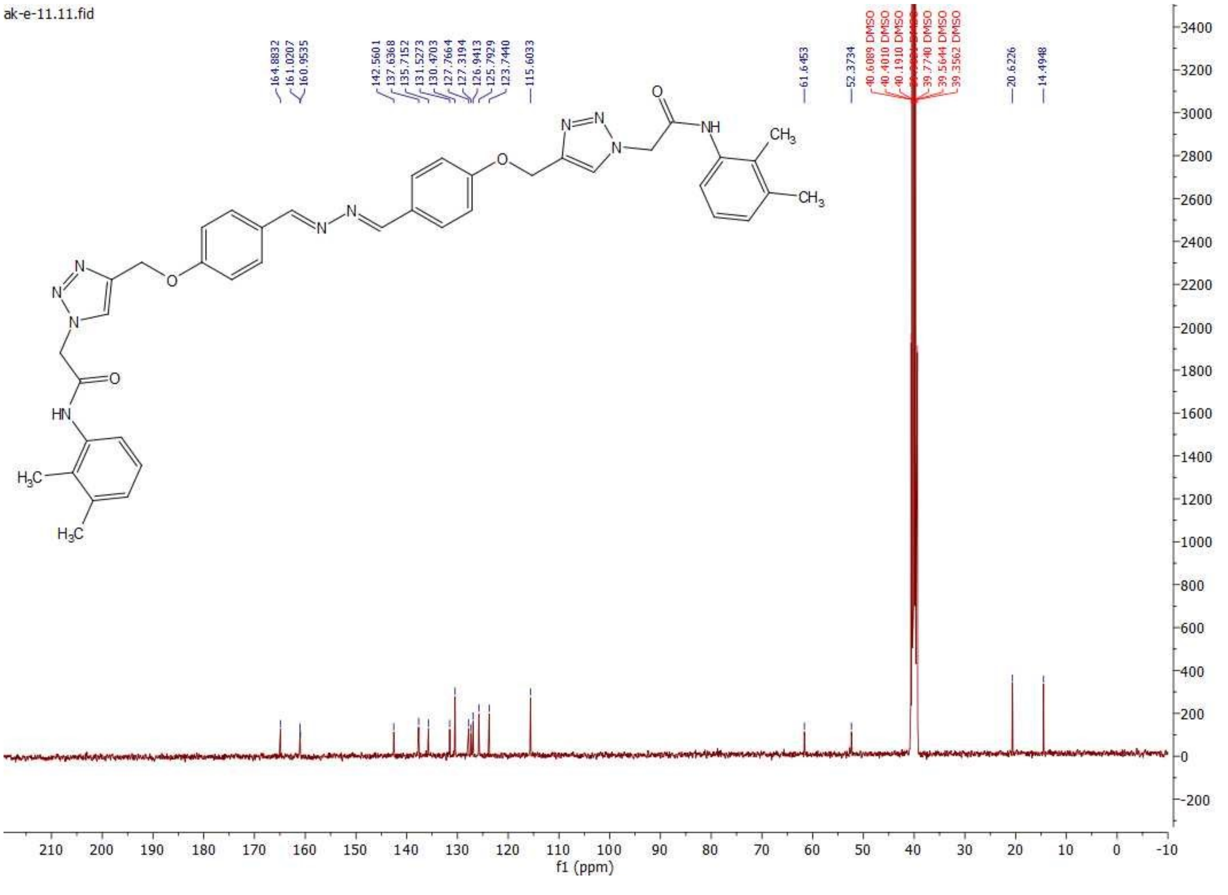

2,2'-((((((1E,1'E)-hydrazine-1,2-diylidenebis(methaneylylidene))bis(4,1-phenylene))bis(oxy))bis(methylene))bis(1H-1,2,3-triazole-4,1-diyl))bis(N-(2,4-dimethoxyphenyl)acetamide) (10e)

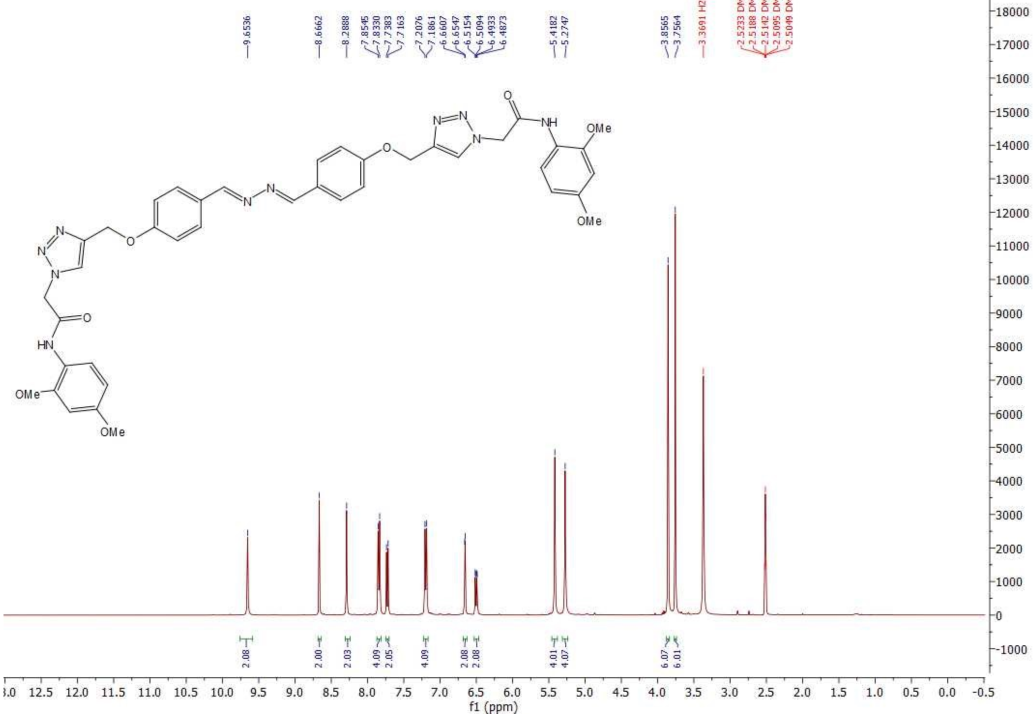

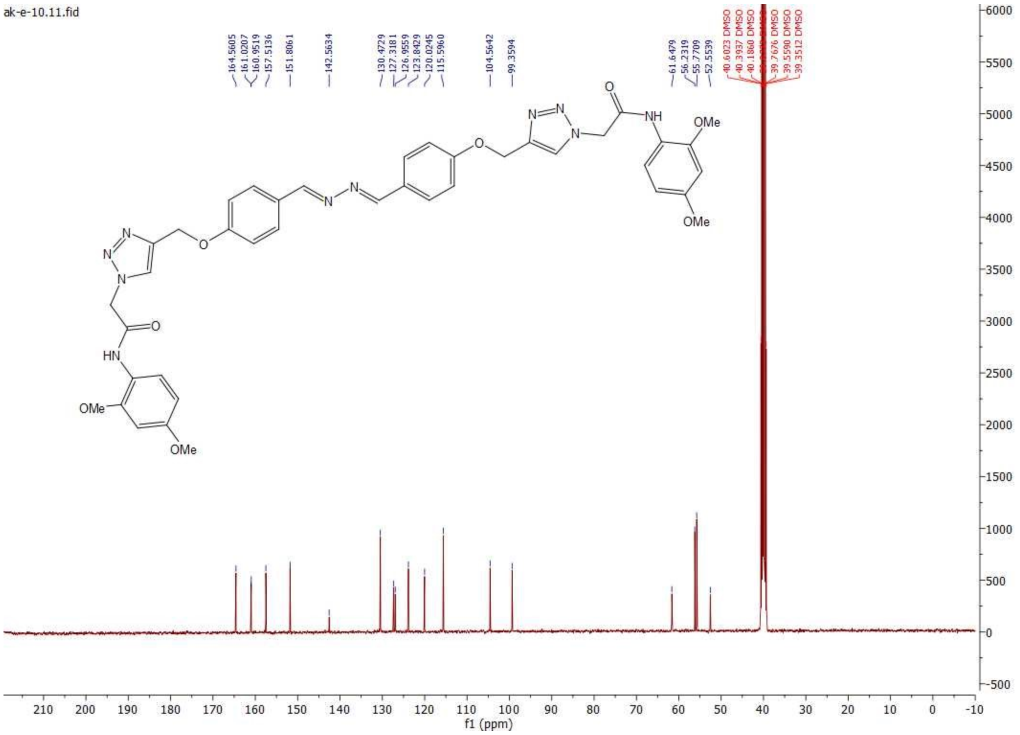

2,2'-((((((1E,1'E)-hydrazine-1,2-diylidenebis(methaneylylidene))bis(4,1-phenylene))bis(oxy))bis(methylene))bis(1H-1,2,3-triazole-4,1-diyl))bis(N-(4-ethylphenyl)acetamide) (10f)

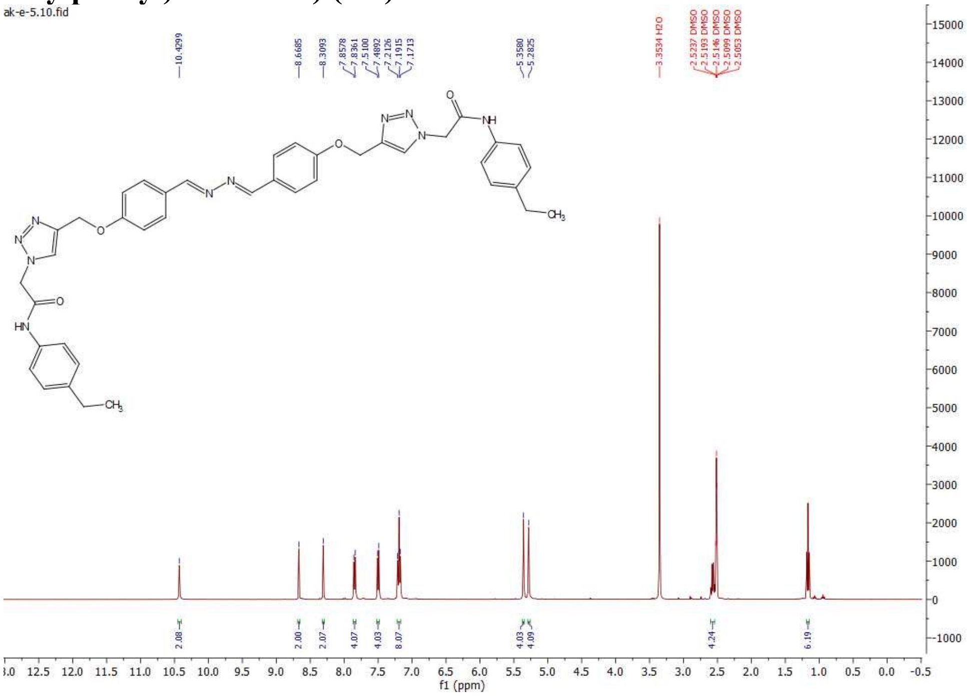

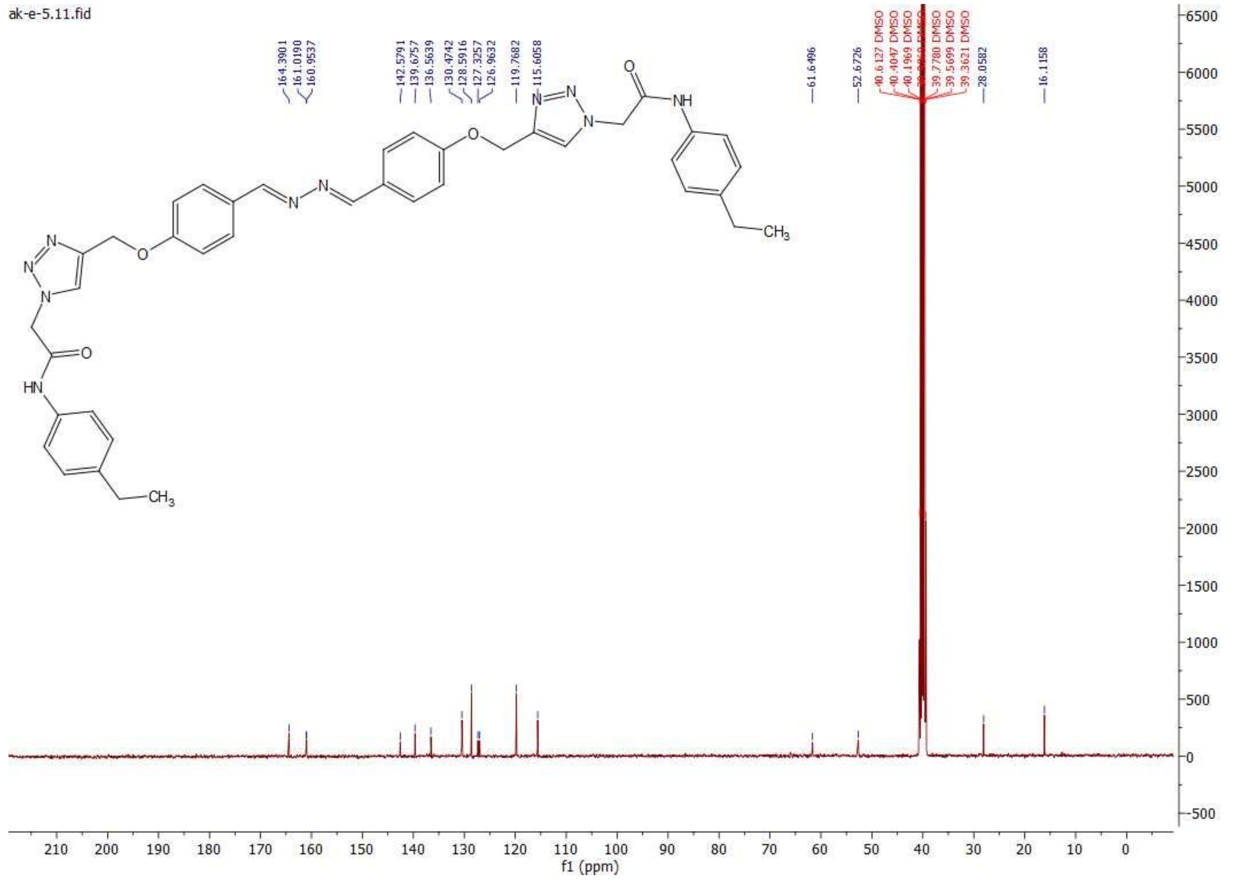

2,2'-((((((1E,1'E)-hydrazine-1,2-diylidenebis(methaneylylidene))bis(4,1-phenylene))bis(oxy))bis(methylene))bis(1H-1,2,3-triazole-4,1-diyl))bis(N-(2-fluorophenyl)acetamide) (10g)

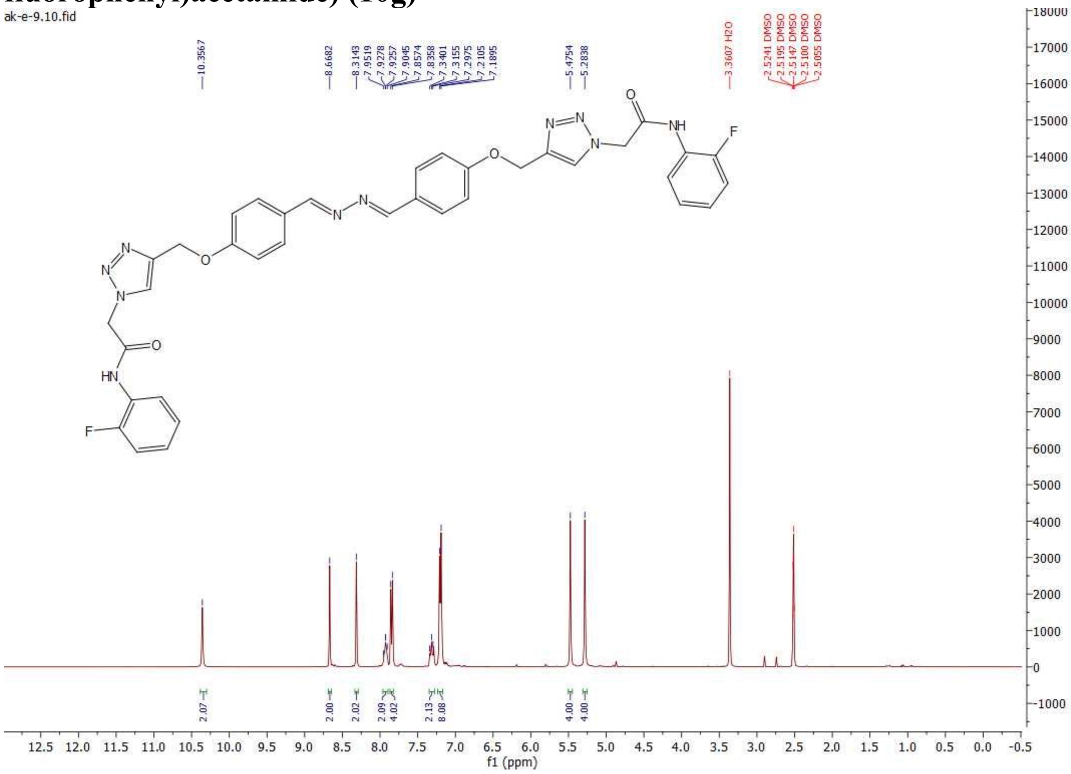

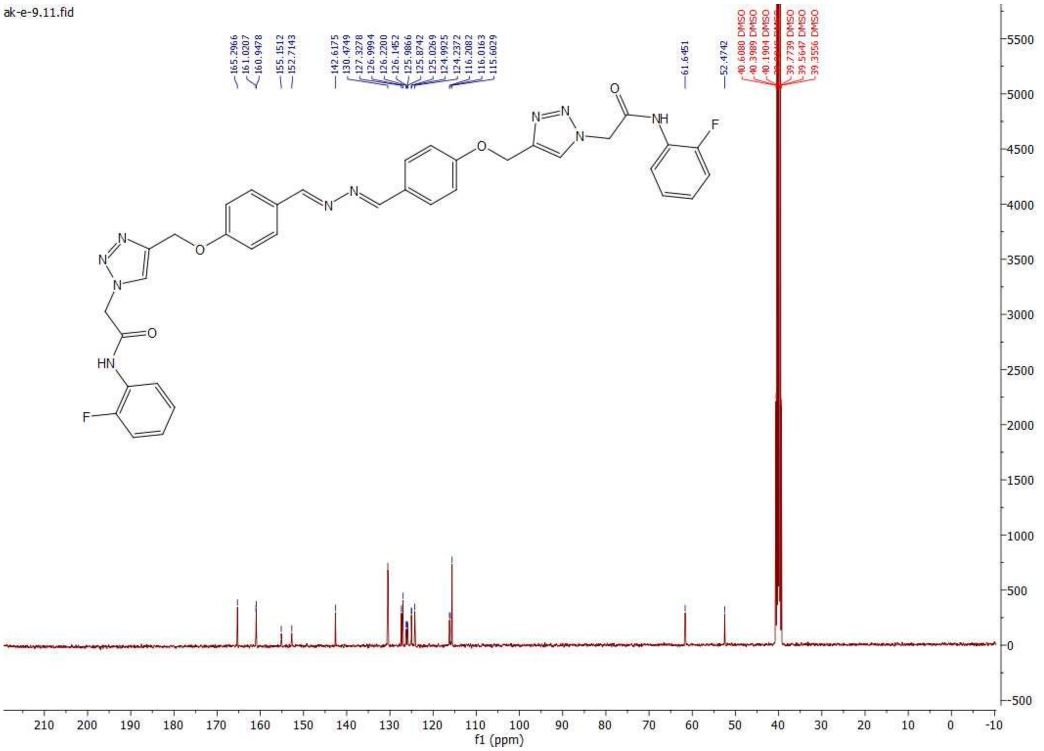

2,2'-((((((1E,1'E)-hydrazine-1,2-diylidenebis(methaneylylidene))bis(4,1-phenylene))bis(oxy))bis(methylene))bis(1H-1,2,3-triazole-4,1-diyl))bis(N-(2-chlorophenyl)acetamide) (10h)

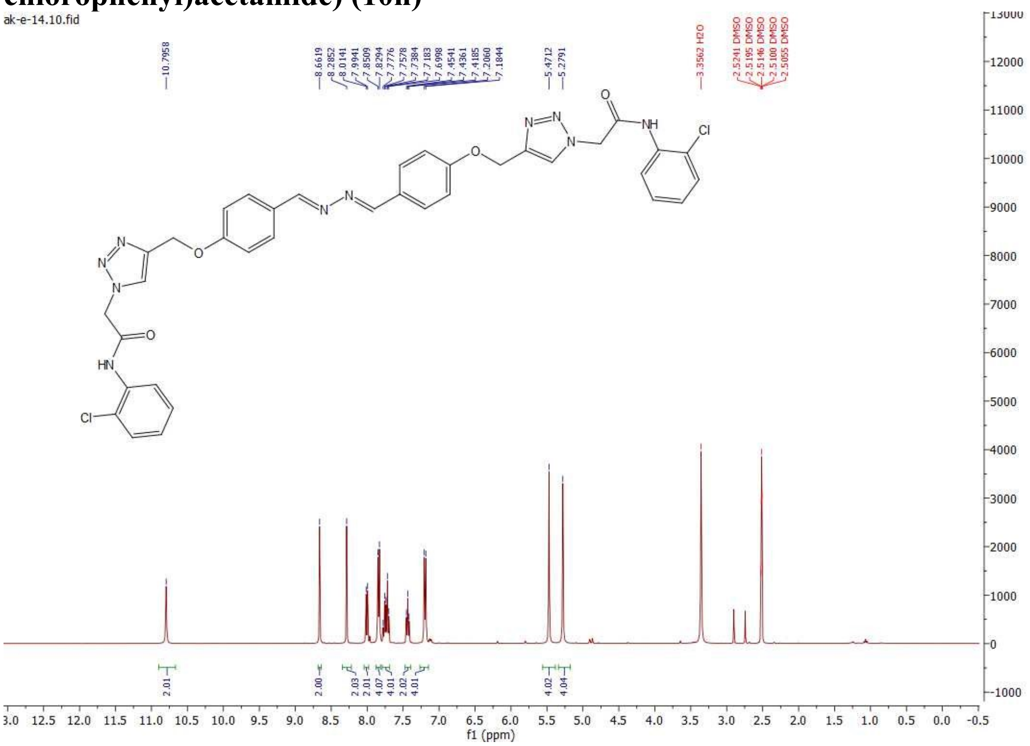

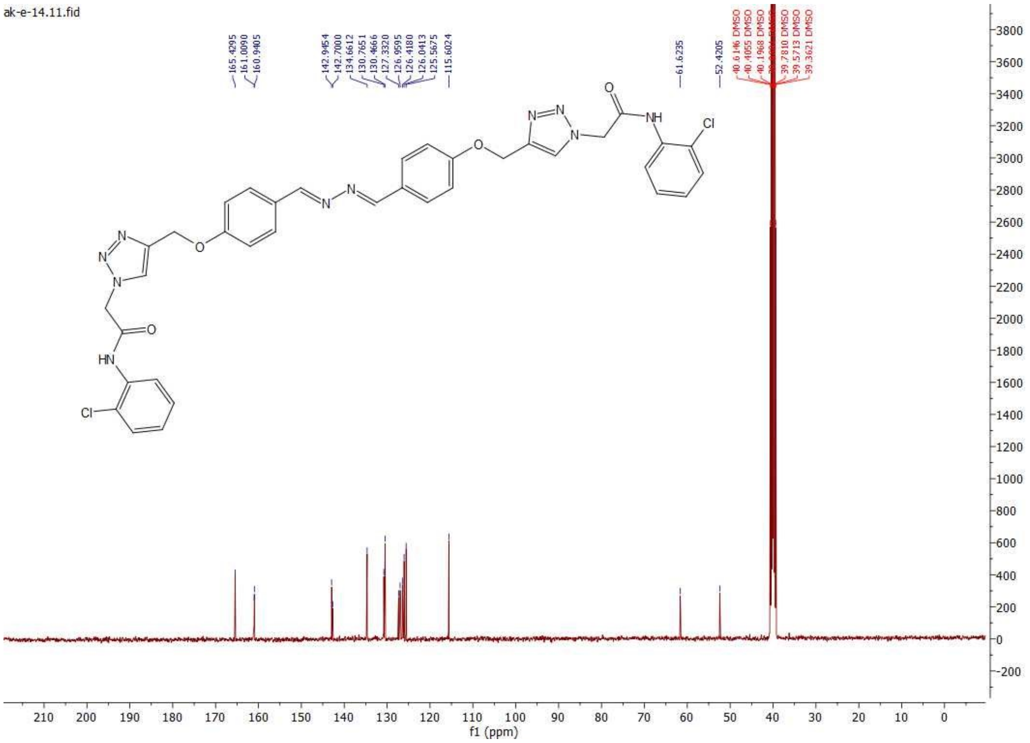

2,2'-((((((1E,1'E)-hydrazine-1,2-diylidenebis(methaneylylidene))bis(4,1-phenylene))bis(oxy))bis(methylene))bis(1H-1,2,3-triazole-4,1-diyl))bis(N-(3-chlorophenyl)acetamide) (10i)

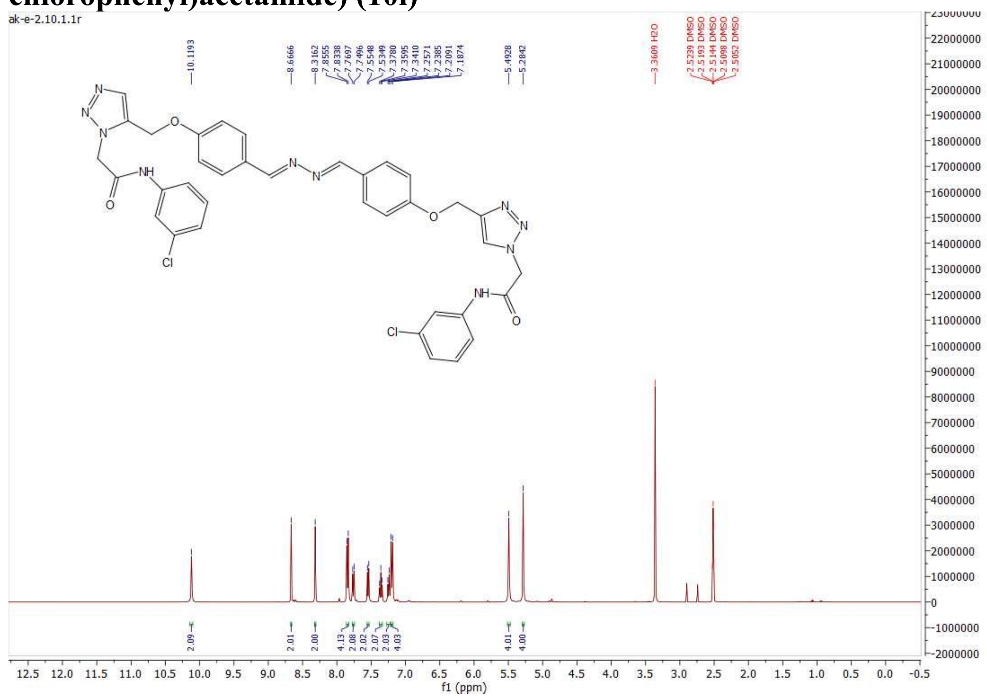

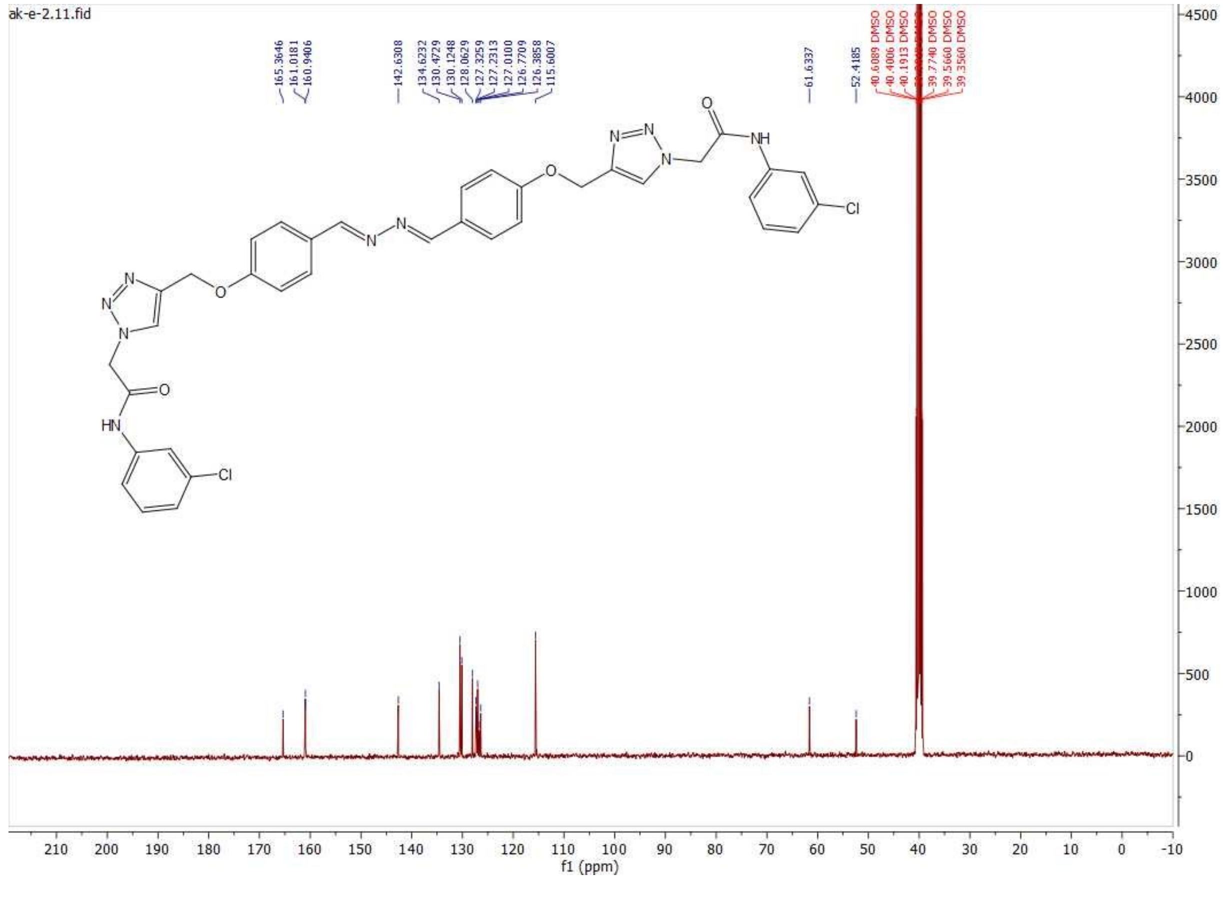

2,2'-((((((1E,1'E)-hydrazine-1,2-diylidenebis(methaneylylidene))bis(4,1-phenylene))bis(oxy))bis(methylene))bis(1H-1,2,3-triazole-4,1-diyl))bis(N-(4-chlorophenyl)acetamide) (10j)

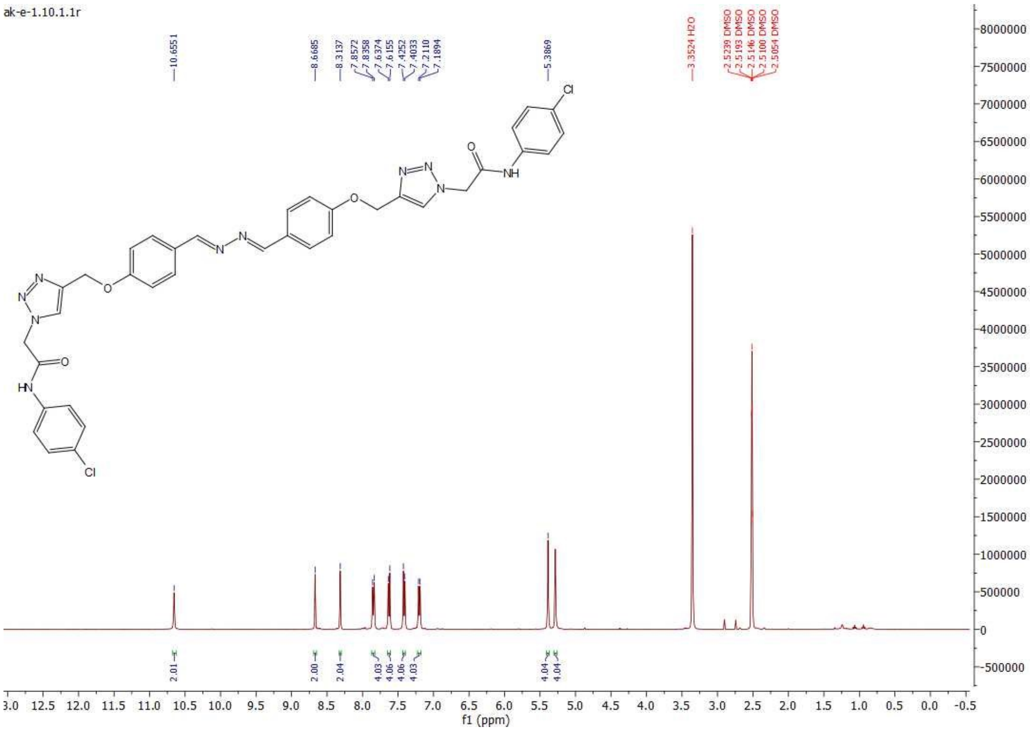

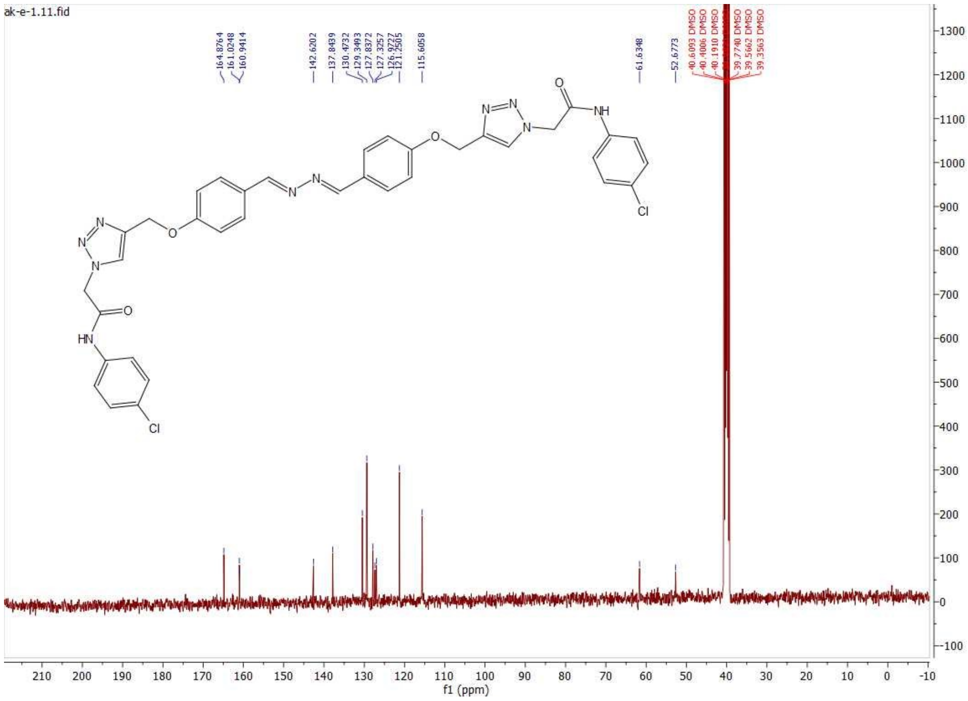

2,2'-((((((1E,1'E)-hydrazine-1,2-diylidenebis(methaneylylidene))bis(4,1-phenylene))bis(oxy))bis(methylene))bis(1H-1,2,3-triazole-4,1-diyl))bis(N-(2,4-dichlorophenyl)acetamide) (10k)

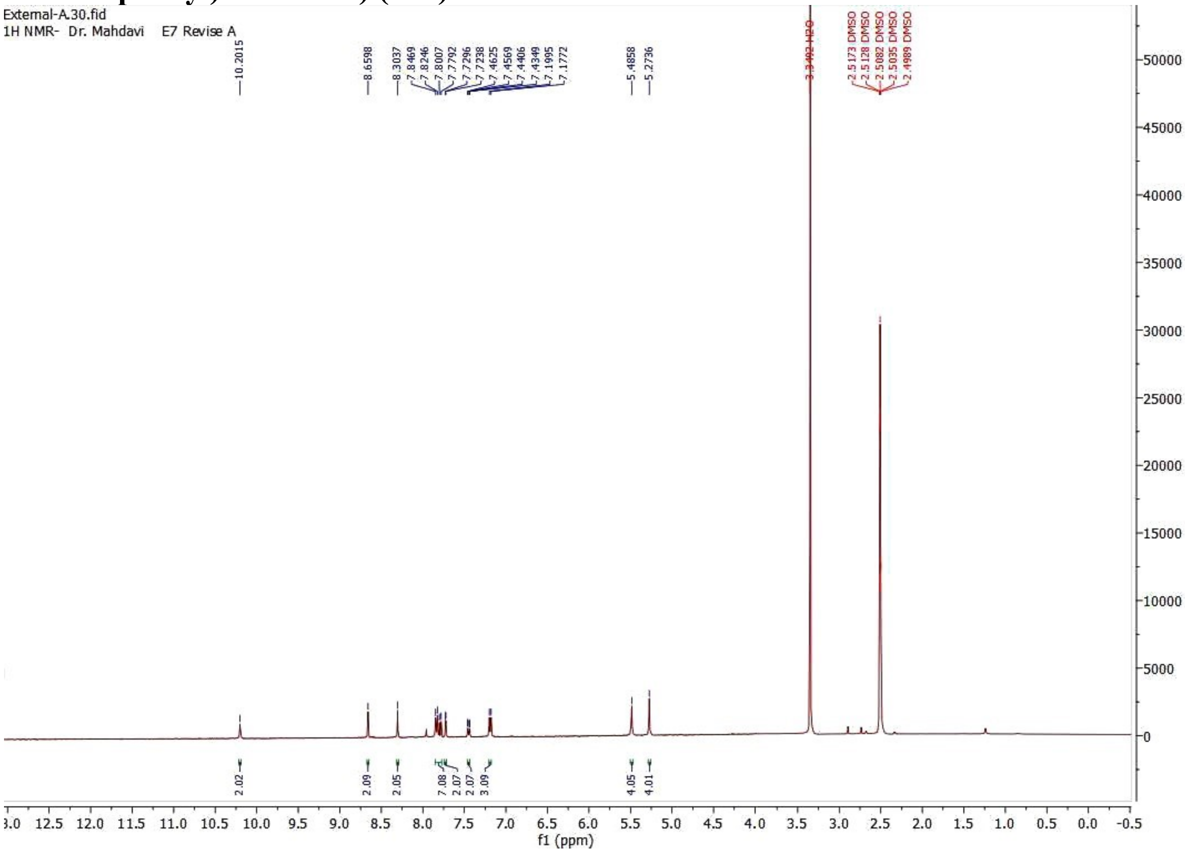

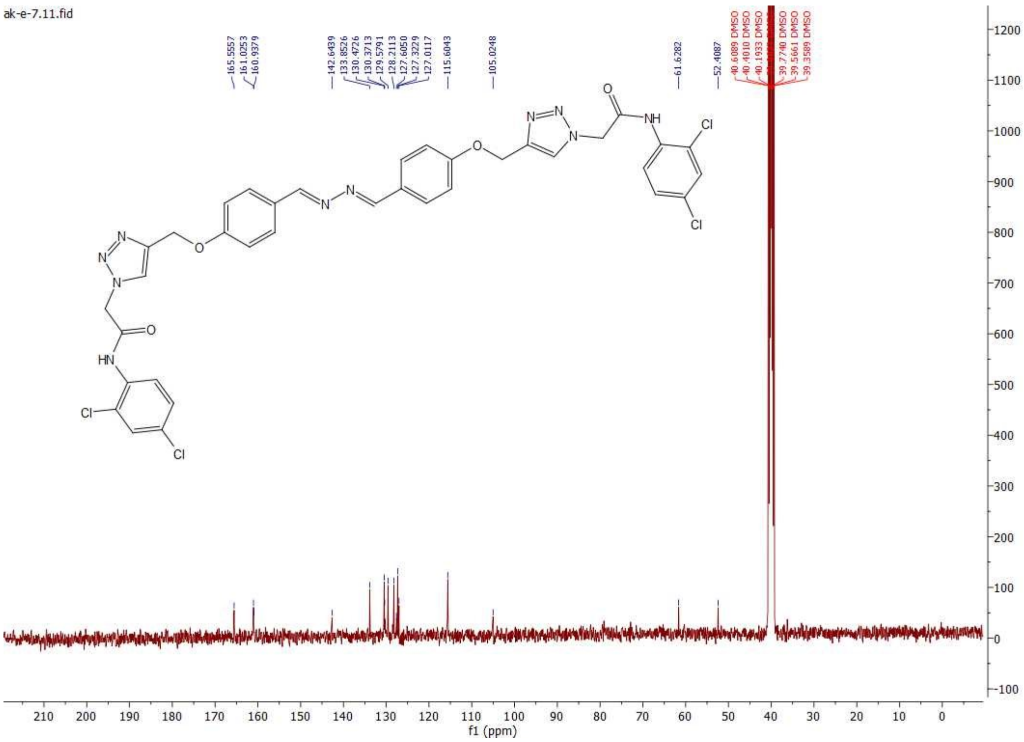

2,2'-((((((1E,1'E)-hydrazine-1,2-diylidenebis(methaneylylidene))bis(4,1-phenylene))bis(oxy))bis(methylene))bis(1H-1,2,3-triazole-4,1-diyl))bis(N-(4-bromophenyl)acetamide) (10l)

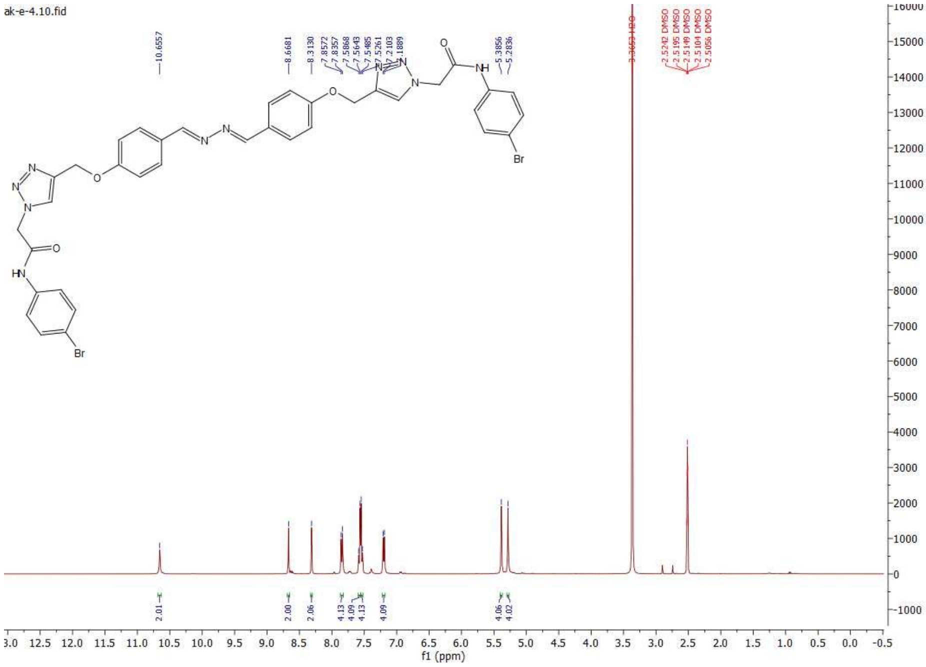

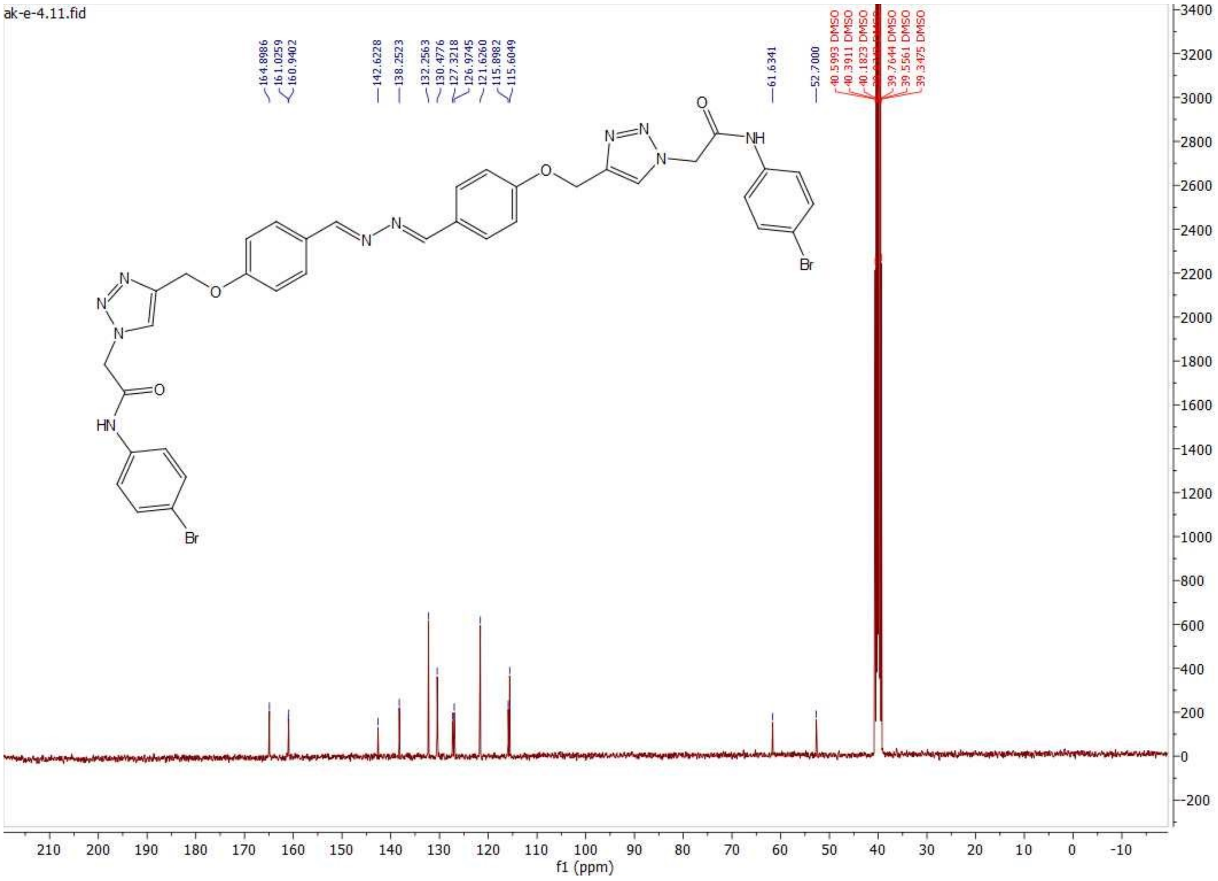

2,2'-((((((1E,1'E)-hydrazine-1,2-diylidenebis(methaneylylidene))bis(4,1-phenylene))bis(oxy))bis(methylene))bis(1H-1,2,3-triazole-4,1-diyl))bis(N-(2-nitrophenyl)acetamide) (10m)

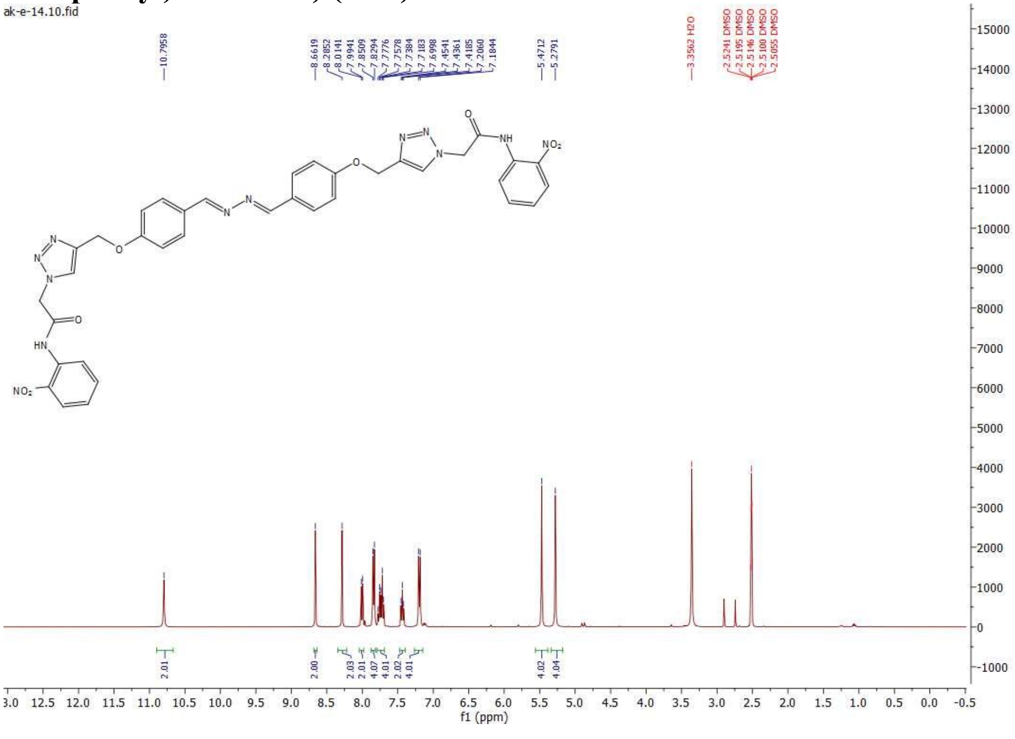

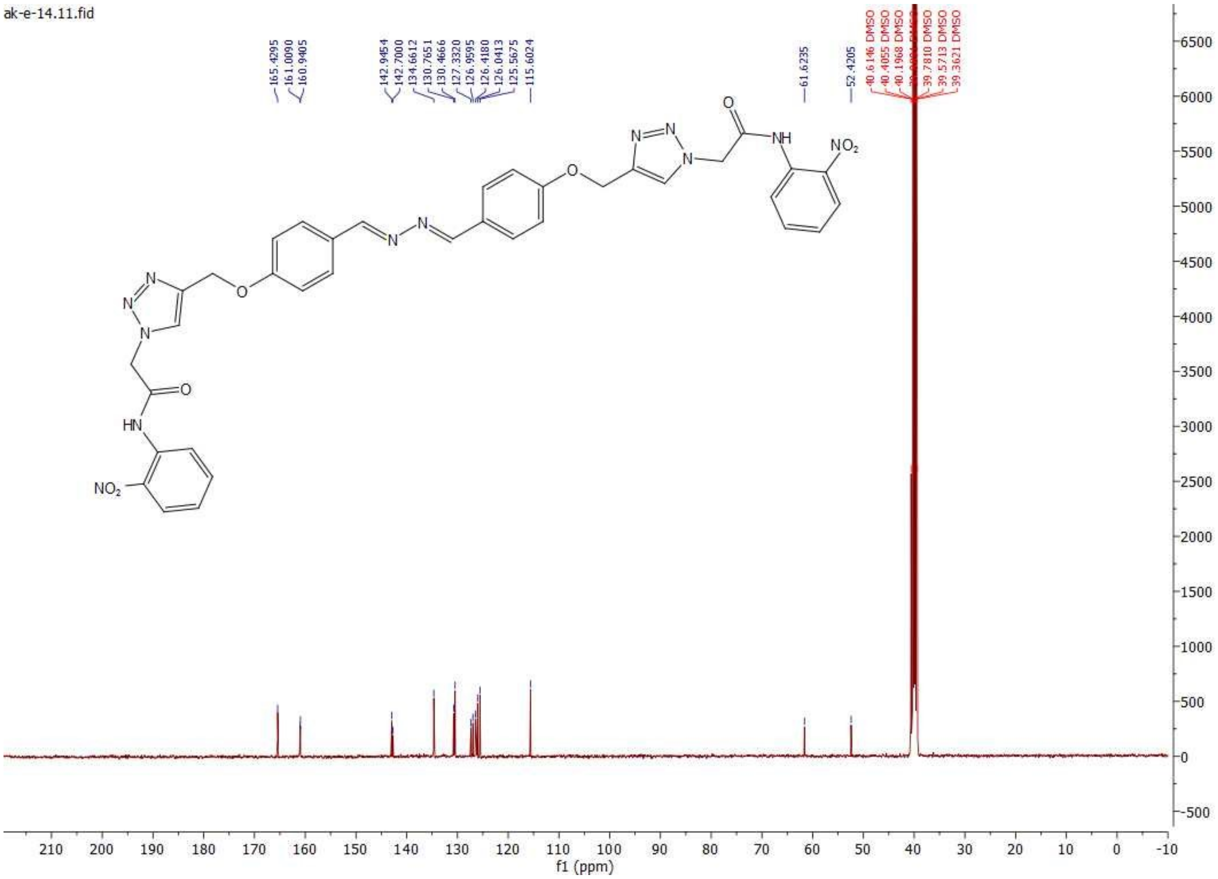

2,2'-((((((1E,1'E)-hydrazine-1,2-diylidenebis(methaneylylidene))bis(4,1-phenylene))bis(oxy))bis(methylene))bis(1H-1,2,3-triazole-4,1-diyl))bis(N-(4-nitrophenyl)acetamide) (10n)

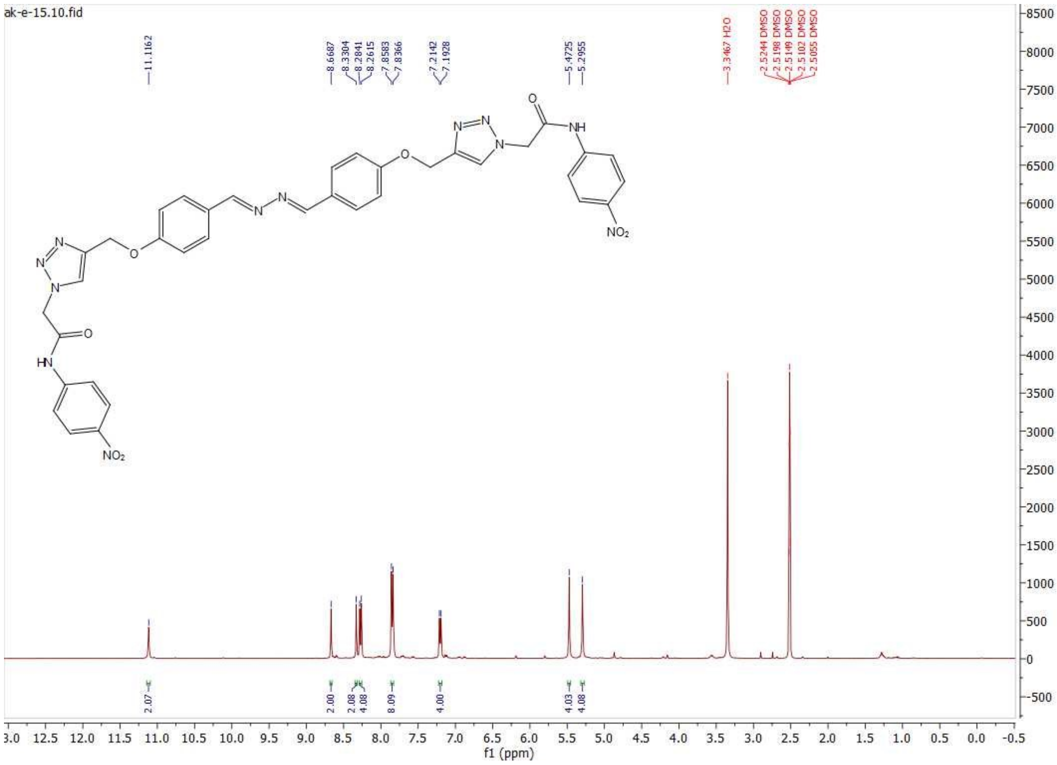
ak-e-15.10.fid
11.1162
8.6687
8.3304
8.2841
8.2615
7.8583
7.8366
7.2142
7.1928
5.4725
5.2955
3.3467 H2O
2.5244 DMSO
2.5198 DMSO
2.5149 DMSO
2.5102 DMSO
2.5055 DMSO
2.07
2.00
2.08
4.08
8.09
4.00
4.03
4.08
f1 (ppm)

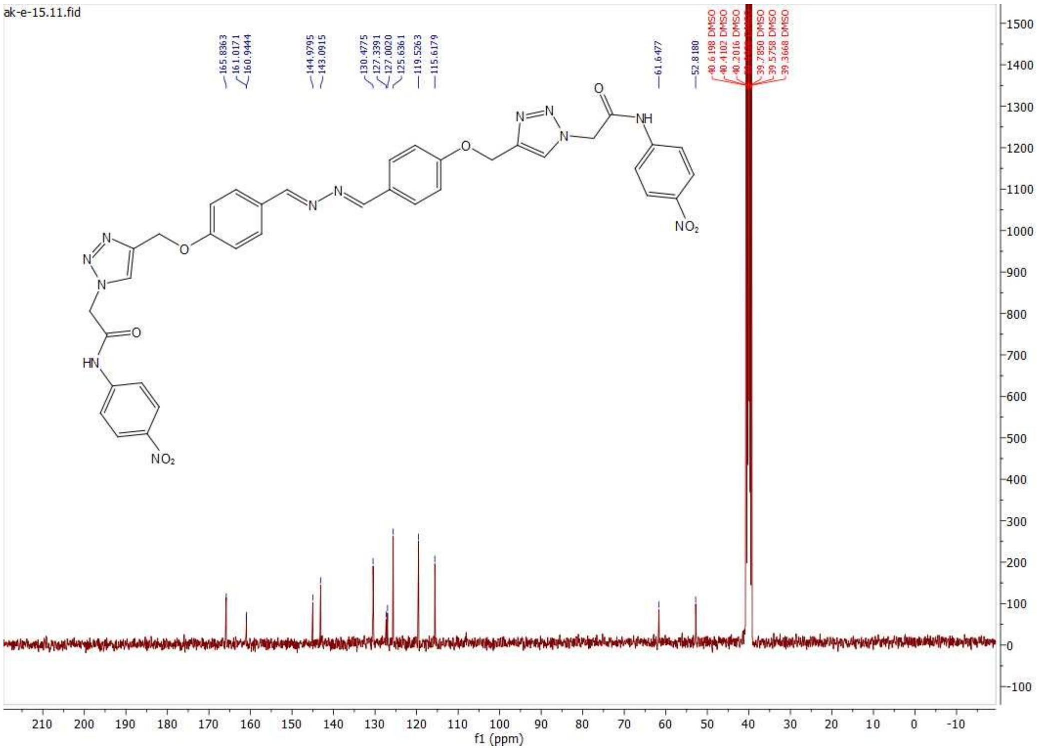
ak-e-15.11.fid
165.8363
161.0171
160.9444
144.9795
143.0915
130.4775
127.3391
127.0020
125.6361
119.5263
115.6179
61.6477
52.8180
40.6198 DMSO
40.4102 DMSO
40.2016 DMSO
39.7850 DMSO
39.5758 DMSO
39.3668 DMSO
f1 (ppm)
